# Growth retardation-responsive analysis of mRNAs and long noncoding RNAs in the liver tissue of Leiqiong cattle

**DOI:** 10.1038/s41598-020-71206-4

**Published:** 2020-08-31

**Authors:** Lingxuan Kong, Guangbin Liu, Ming Deng, Zhiquan Lian, Yinru Han, Baoli Sun, Yongqing Guo, Dewu Liu, Yaokun Li

**Affiliations:** 1 College of Animal Science, South China Agricultural University, Guangzhou, 510642 GD China; 2 National Local Joint Engineering Research Center of Livestock and Poutry, South China Agricultural University, Guangzhou, 510642 GD China

**Keywords:** Animal breeding, Gene expression, Genomics

## Abstract

As an important type of non-coding RNA molecule, long non-coding RNAs (lncRNAs) have varied roles in many biological processes, and have been studied extensively over the past few years. However, little is known about lncRNA-mediated regulation during cattle growth and development. Therefore, in the present study, RNA sequencing was used to determine the expression level of mRNAs and lncRNAs in the liver of adult Leiqiong cattle under the condition of growth retardation and normal growth. We totally detected 1,124 and 24 differentially expressed mRNAs and lncRNAs, respectively. The differentially expressed mRNAs were mainly associated with growth factor binding, protein K63-linked ubiquitination and cellular protein metabolic process; additionally, they were significantly enriched in the growth and development related pathways, including PPAR signaling pathway, vitamin B6 metabolism, glyoxylate and dicarboxylate metabolism. Combined analysis showed that the co-located differentially expressed lncRNA Lnc_002583 might positively influence the expression of the corresponding genes IFI44 and IFI44L, exerting co-regulative effects on Leiqiong cattle growth and development. Thus, we made the hypothesis that Lnc_002583, IFI44 and IFI44L might function synergistically to regulate the growth of Leiqiong cattle. This study provides a catalog of Leiqiong cattle liver mRNAs and lncRNAs, and will contribute to a better understanding of the molecular mechanism underlying growth regulataion.

## Introduction

Beef is an excellent source of protein with lower level of lipid content and is rich in Fe, P and Zn. Leiqiong cattle is one of the typical representative cattle in South China. It is heat-resistant, crude feed-resistant and has strong resistance to disease. However, Leiqiong cattle is small in size and low in meat production, which can not meet the needs of the market. Moreover, in the actual livestock production, some Leiqiong cattle will always have growth retardation. Currently, the causes of animal growth retardation are mainly divided into four categories, namely genetics, nutrition, disease, and feeding management. Animal growth retardation can cause a series of problems: the quality of the meat produced will decrease, the milk yield of dairy animals will decrease or milk production could cease, and the quality and grade of the fur of fur-producing animals will decrease. Thus, the economic benefits of animal production will be reduced when the animals suffer retardation.

Currently, research on animal growth retardation has focused on hormonal regulation and nutritional regulation. Japanese Black Cattle with growth retardation showed lower condentrations of serum growth hormone (GH) and serum insulin like growth factor 1 (IGF1), suggesting defects in the GH-IGF1 axis^1^. Studies showed that GH deficient mice are 40% lighter than normal mice; IGF1 receptor deficient mice are 55% lighter than normal mice; meanwhile, these genetic deficient animals growth rate is significantly reduced, and the development of muscle and bone is also inhibited^2^. Additionally, it was suggested that daily feeding of 70 mg/kg body weight of cysteamine can significantly increase the daily weight gain and serum IGF1 levels of growing and finishing pigs, and can upregulate the expression of the growth hormone receptor (GHR), IGF1, IGF1 receptor (IGF1R), and insulin like growth factor binding protein 3 (IGFBP3) genes in multiple tissues^3^. The supplementation of sucrose can also improve the growth performance of slow-growing calves^4^. During feeding process, the growth retardation yak was supplemented with sucrose (1 g/kg body weigh) twice a week; compared with growth-stasis cattle without sucrose supplementation, the body weight of sucrose-added yak was significantly increased and was similar to market weight; it was further depicted that the sucrose could induced rumen nipple development, which facilitated the compensatory growth of retardation yak. Genetic studies on growth retardation showed that if the homozygous form of the mouse Akt1 gene was disrupted, the mice would experience growth retardation^5^. Mice knocked out of both Akt1 and Akt2 showed weight loss, skeletal muscle atrophy, decreased fat deposition, and growth retardation^6^. When IGF1R was mutated, humans would suffer growth retardation^7^. The knockout of TMEFF2 gene could also result in growth retardation and severe loss of white adipose tissue in mice^8^. For growth retardation piglets, INS, IGF-1, and phosphorylation of AKT were significantly down-regulated^9^. Studies on animal growth retardation have focused on intrauterine growth retardation (IUGR). The IUGR phenomenon, for example, is widespread among multiparous animals and even among populations in many countries, with about 10% of babies born in the U.S. being affected by IUGR^10^. So far, animals such as rats^11^, mice^10^, guinea pigs^12^, white rabbits^13^, sheep^14^, dogs^15^, and pigs^13^ have been used to build IUGR models. IUGR not only affects the animal's birth weight and organ weight, but also affects the growth and development of the body and organs of the newborn after birth, such as the brain, small intestine, liver, muscles and so on^9,16^. It also affects the metabolism of animal liver nutrients such as carbohydrates, fatty acids, and amino acid metabolism^16,17^. Fatty acid beta oxidation in growth-stable newborn piglets is an important pathway for energy production, and some protein expressions are significantly down-regulated with fatty acid beta oxidation (EHHADH, GCDH and ACOX2), cholesterol synthesis (LSS), and lipid transport (APOE)^18–20^. Thus, we decided to study the molecular mechanisms that affect growth retardation in Leiqiong cattle. There is almost no research on the combination of long non-coding RNAs (lncRNAs) and growth retardation, and there is also little research on cattle growth retardation. The liver is the crucial metabolic organ associated with the growth response, and little is known about the mechanisms underlying growth retardation and development in cattle. Therefore, we focused on transcriptome analysis of the livers of growth retarded Leiqiong cattle and normal growth Leiqiong cattle to identify growth-related mRNAs, lncRNAs, and regulatory pathways. We hypothesized that this approach would identify only the critical genes and pathways involved in the growth retardation response. The genes identified in this study could serve as candidates for breeding Leiqiong cattle with superior growth performance.

## Methods

### Animals and sample collection

The Leiqiong cattle (genus: *Bos*; species: *Bos indicus*) were born, raised, and maintained at the Guangdong Leiqiong cattle farm. In this study, all animals were healthy and received the same diet until they were slaughtered in adulthood (24 months). The experimental animals were divided into two groups: growth retardation cattle (GRC; the average body height was 95 cm, and the average live weight before slaughter was 153.5 kg) and normal growth cattle (NGC; the average body height was 106 cm, and the average live weight before slaughter was 206.7 kg). All the experimental animals were without any nutritional manipulations; they were simply spontaneously different in size and weight. Liver samples at the same pre-determined site were collected from GRC and NGC (n = 3 at each group, female) and immediately frozen in liquid nitrogen for total RNA extraction after harvesting.

### cDNA library preparation

We isolated the total RNA by using the TRIzol reagent (Invitrogen, Carlsbad, CA, USA) and DNase I (Qiagen, Beijing, China). Then, 1.5% agarose gel electrophoresis was used to assess the quality of the purified RNA, confirming the absence of genomic DNA. The RNA integrity was estimated using an RNA Nano6000 Assay Kit and a Bioanalyzer 2100 system (Agilent Technologies, Santa Clara, CA, USA)^21^.

For the cDNA library contruction, 3 μg of total RNA (the value of RNA integrity number was all larger than 7.0) was utilized as the input for each sample. Firstly, we discarded the ribosomal RNA (rRNA) through the Epicentre Ribo-zero rRNA Removal Kit (Epicentre, Madison, WI, USA), the rRNA-free residue was cleaned by ethanol precipitation. Next, sequencing libraries were constructed using the rRNA-deleted RNA with a NEBNext Ultra Directional RNA Library Prep Kit for Illumina (NEB, Ipswich, MA, USA) referring to the manufacturer’s instructions. After fragmentation in NEBNext First Strand Synthesis Reaction Buffer (5 ×), we subsequently synthesized the first strand and second strand cDNA. After adenylation of the 3′ ends of the DNA fragments, NEBNext Adaptors containing a hairpin loop structure were ligated to prepare for hybridization. The library fragments were purified using an AMPure XP system (Beckman Coulter, Beverly, MA, USA), preferentially selecting the cDNA fragments of 150–200 bp in length. Then, USER enzyme (NEB) was utilized to incubate with the size-selected, adaptor-ligated cDNA at 37 °C for 15 min followed by 5 min at 95 °C. PCR amplification was then performed using Phusion High-Fidelity DNA polymerase, Universal PCR primers, and Index (X) Primer. Finally, the products were purified (AMPure XP system) and the library quality was assessed using the Agilent Bioanalyzer 2100 system^21^.

### Sequencing and transcriptome assembly

The libraries constructed were sequenced on an Illumina HiSeq 4000 platform and 150-bp paired-end reads were generated. After removing sequences containing adapters and the reads containing poly-N and low quality reads using in-house Perl scripts, clean data were obtained. All the downstream analyses were based on the high quality, clean data. To obtain the high quality reads, we performed the following filtering process: removing the reads containing more than 10% unknown nucleotides and the reads containing more than 50% low quality nucleotides with a Phred quality less than 20. Mapping to the reference genome was the next step. Reads that passed the quality control were then mapped to the Bovine reference genome (Bos_taurus.UMD3.1; Ensemble release 94). An index of the reference genome was built using bowtie2 v2.2.8 and paired-end clean reads were aligned to the reference genome using HISAT2 (v2.0.4)^22^. HISAT2 was run with ‘–rna-strandness RF’; other parameters were set as default. Next, transcriptome assembly was performed using the mapped reads of each sample and StringTie (v1.3.1)^23^.

### Prediction of differentially expressed long non-coding RNAs and protein-coding RNAs

Before screening, Cuffmerge was used to create the set of transcripts. Then, lncRNA screening was carried out using the following steps: Step 1: Select the number of exon ≥ 2 transcripts; Step 2: From the results from step 1, select the transcripts with a length > 200 bp; Step 3: Annotate the above transcripts using Cuffcompare software; Step 4: Calculate the expression level of each transcript using Cuffquant, and select transcripts with a fragments per kilo base of exon per million fragments mapped (FPKM) value ≥ 0.1; Step 5: Coding potential screening. The coding potential of the transcript was predicted by three software modules: CNCI (Coding-Non-Coding-Index) (v2)^24^, CPC (Coding Potential Calculator) (0.9-r2)^25^, and PFAM (Protein Families Database; Pfam Scan, v1.3)^26,27^; the intersection of transcripts without coding potential screened using the above three software modules with default parameters was predicted as the lncRNA dataset.

The Ballgown software was used to perform the straightforward linear-model-based differential expression analyses with the default statistical modeling framework^23,28^. Transcripts with an adjusted *P* value < 0.05 were assigned as differentially expressed.

### Target gene prediction of the lncRNAs

For each lncRNA locus, the 100-kb regions upstream and downstream were chosen to screen for the cis-acting target genes using the UCSC Genome Bioinformatics tool, which were also named co-located genes.

### GO and KEGG enrichment analysis

Gene Ontology (GO) enrichment analysis of differentially expressed genes (DEGs) or lncRNA target genes was implemented using the GOseq R package, in which gene length bias was corrected^29^. GO terms with corrected *P* < 0.05 were considered significantly enriched for DEGs.

Kyoto Encyclopedia of Genes and Genomes (KEGG) is a database resource for understanding high-level functions and utilities of biological systems^30^, such as the cell, organism and ecosystem, from molecular-level information, especially large-scale molecular datasets generated by genome sequencing and other high-throughput experimental technologies (https://www.genome.jp/kegg/). We used KOBAS software to analyze the enrichment of DEGs or lncRNA target genes in KEGG pathways^31^.

### Quantitative real-time reverse transcription polymerase chain reaction validation

For the quantitative real-time reverse transcription polymerase chain reaction (qRT-PCR) analysis, 1 µg of total RNA was reverse transcribed using an RT reagent Kit with gDNA Eraser (Takara, Dalian, China) according to the manufacturer’s protocol. The qRT-PCR reactions were performed on a StepOnePlus Real-Time PCR System (Life Technologies, Carlsbad, CA, USA) according to standard methods using Fast Start Universal SYBR Green Master (ROX) (Roche, Mannheim, Germany).

### Ethics approval and consent to participate

The study was approved by the Ethics Committees of Laboratory Animal Center of South China Agricultural University (permit number: SYXK-2014-0136). All experiments were performed in accordance with South China Agricultural University guidelines.

## Results

### Read mapping

For RNA-seq data analysis, the proportion of the nucleotides with Q30 in the total nucleotides should be generally larger than 85%; in our study, the proportion of each sample exceeded 91%. In total, 89,186,385, 77,069,075, 80,073,002, 91,238,926, 94,484,701 and 89,487,657 mapped reads were obtained from the clean data from the GRC1, GRC2, GRC3, NGC4, NGC5, and NGC6 libraries, respectively, and more than 94% were mapped to the Bovine reference genome (Table 1).

**Table 1:** Summary of clean reads mapped to the *Bovine* reference genome.

Sample	GRC1	GRC2	GRC3	NGC4	NGC5	NGC6
Raw reads	97,059,272	84,633,280	87,684,370	99,905,226	103,732,518	97,539,080
Clean reads	94,190,850	81,529,150	85,130,830	96,699,986	100,268,668	94,588,436
Q30 (%)	93.39	93.39	91.98	93.36	93.48	93.44
GC content (%)	49.29	50.38	50.06	50.22	49.78	48.77
Total mapped	89,186,385 (94.69%)	77,069,075 (94.53%)	80,073,002 (94.06%)	91,238,926 (94.35%)	94,484,701 (94.23%)	89,487,657 (94.61%)

*GRC* growth retardation cattle, *NGC* normal growth cattle.

### Enrichment analysis of differentially expressed mRNAs

**Figure 1:**
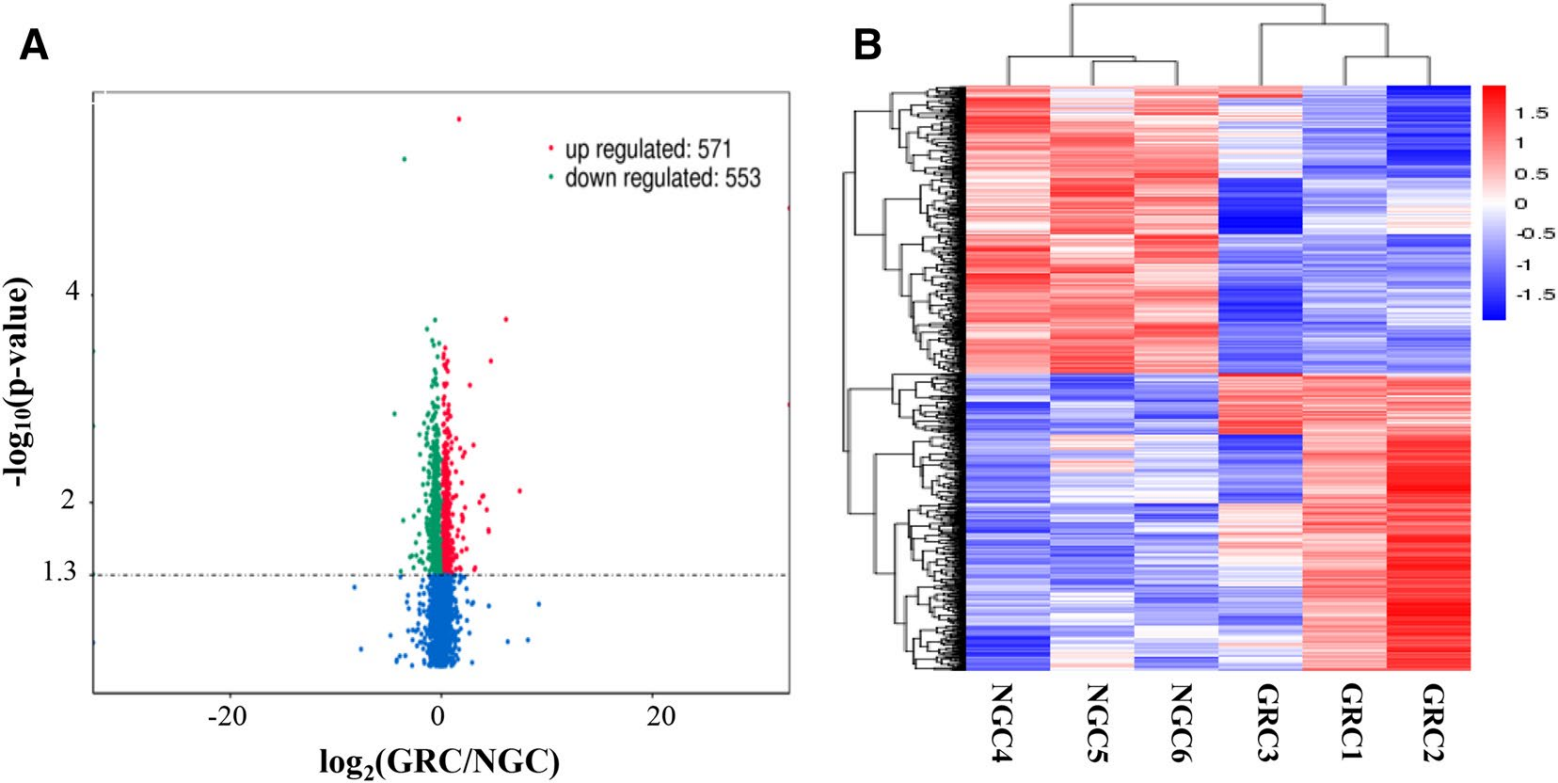
Analyses of differentially expressed mRNAs in the RNA sequencing (RNA-seq) libraries. (**A**) Volcano plot showing the overall distribution of the differential transcript or gene, with the threshold set to q < 0.05. Red: relatively high expression; Green: relatively low expression. (**B**) Hierarchical clustering analysis of mRNA expression profiles from 11 libraries with 1,124 differentially expressed mRNAs. Data are expressed as fragments per kilo base of exon per million fragments mapped (FPKM). Red: relatively high expression; Blue: relatively low expression.

We totally discovered 1,124 differentially expressed mRNA transcripts. Compared with the NGC group, 571 and 553 mRNAs were respectively upregulated and downregulated in the GRC group (Fig. 1A and Additional file 1). Systematic cluster analysis showed that the expression pattern of the differentially expressed mRNAs was obviously distinct between the two experimental groups (Fig. 1B). To further depict the biological function of the differentially expressed mRNAs, we performed GO and KEGG enrichment analysis. In total, 609 GO terms with *P* < 0.05 were found (Additional file 2); most of them were associated with growth and development, including growth factor binding, glucocorticoid receptor signaling pathway, “protein K63-linked ubiquitination, and cellular protein metabolic process (Table 2). Additionally, we detected 31 KEGG pathways significantly enriched by the differentially expressed mRNAs (*P* < 0.05) (Table 3 and Additional file 3), several of which were related to growth and development, including PPAR signaling pathway, vitamin B6 metabolism, glyoxylate and dicarboxylate metabolism, and fatty acid metabolism. Of the top 50 DEGs, WIF1 was involved in the Wnt signaling pathway, and it also showed association with the regulation of fat cell differentiation, regulation of cell differentiation, and cellular developmental process; SDHAF4 was related to system development, organ development, single-organism developmental process and metabolic process.

**Table 2:** Biological process enriched by the differentially expressed mRNAs.

GO terms	Number of genes	*P* value
**Biological_process**		
GO:0019752: carboxylic acid metabolic process	52	1.56 × 10^–6^
GO:0006082: organic acid metabolic process	54	7.02 × 10^–6^
GO:0043436: oxoacid metabolic process	53	7.74 × 10^–6^
GO:0010033: response to organic substance	95	3.55 × 10^–5^
GO:0008152: metabolic process	433	4.22 × 10^–5^
GO:0044267: cellular protein metabolic process	186	1.24 × 10^–5^
GO:0030163: protein catabolic process	46	2.05 × 10^–4^
GO:0048522: positive regulation of cellular process	168	6.09 × 10^–3^
GO:0006950: response to stress	117	1.57 × 10^–2^
**Cellular_component**		
Extracellular vesicular exosome	149	2.33 × 10^–12^
GO:0044444: cytoplasmic part	337	4.53 × 10^–12^
GO:0032991: macromolecular complex	223	5.89 × 10^–6^
GO:0043234: protein complex	193	4.04 × 10^–5^
GO:0012505: endomembrane system	67	2.58 × 10^–3^
GO:0005783: endoplasmic reticulum	55	1.06 × 10^–2^
**Molecular_function**		
GO:0016491: oxidoreductase activity	49	9.17 × 10^–5^
GO:0005515: protein binding	255	1.35 × 10^–4^
GO:0003723: RNA binding	81	1.53 × 10^–4^
GO:0003824: catalytic activity	255	4.82 × 10^–4^
GO:0036094: small molecule binding	113	7.19 × 10^–4^
GO:1901265: nucleoside phosphate binding	103	2.46 × 10^–3^

**Table 3:** KEGG pathway enrichment analysis of differentially expressed mRNAs.

KEGG pathways	Number of genes	*P* value
Proteasome	19	3.01 × 10^–8^
Metabolic pathways	127	2.49 × 10^–4^
Protein processing in endoplasmic reticulum	26	8.49 × 10^–4^
Vitamin B6 metabolism	4	6.94 × 10^–3^
Carbon metabolism	16	7.80 × 10^–3^
Glycine, serine and threonine metabolism	9	8.18 × 10^–3^
Tryptophan metabolism	9	1.05 × 10^–2^
Non-alcoholic fatty liver disease (NAFLD)	21	1.32 × 10^–2^
Pyrimidine metabolism	15	1.38 × 10^–2^
Glyoxylate and dicarboxylate metabolism	6	1.71 × 10^–2^
Alanine, aspartate and glutamate metabolism	7	2.27 × 10^–2^
PPAR signaling pathway	11	2.37 × 10^–2^
beta-Alanine metabolism	6	4.28 × 10^–2^

### Enrichment analysis of the differentially expressed lncRNAs

**Figure 2:**
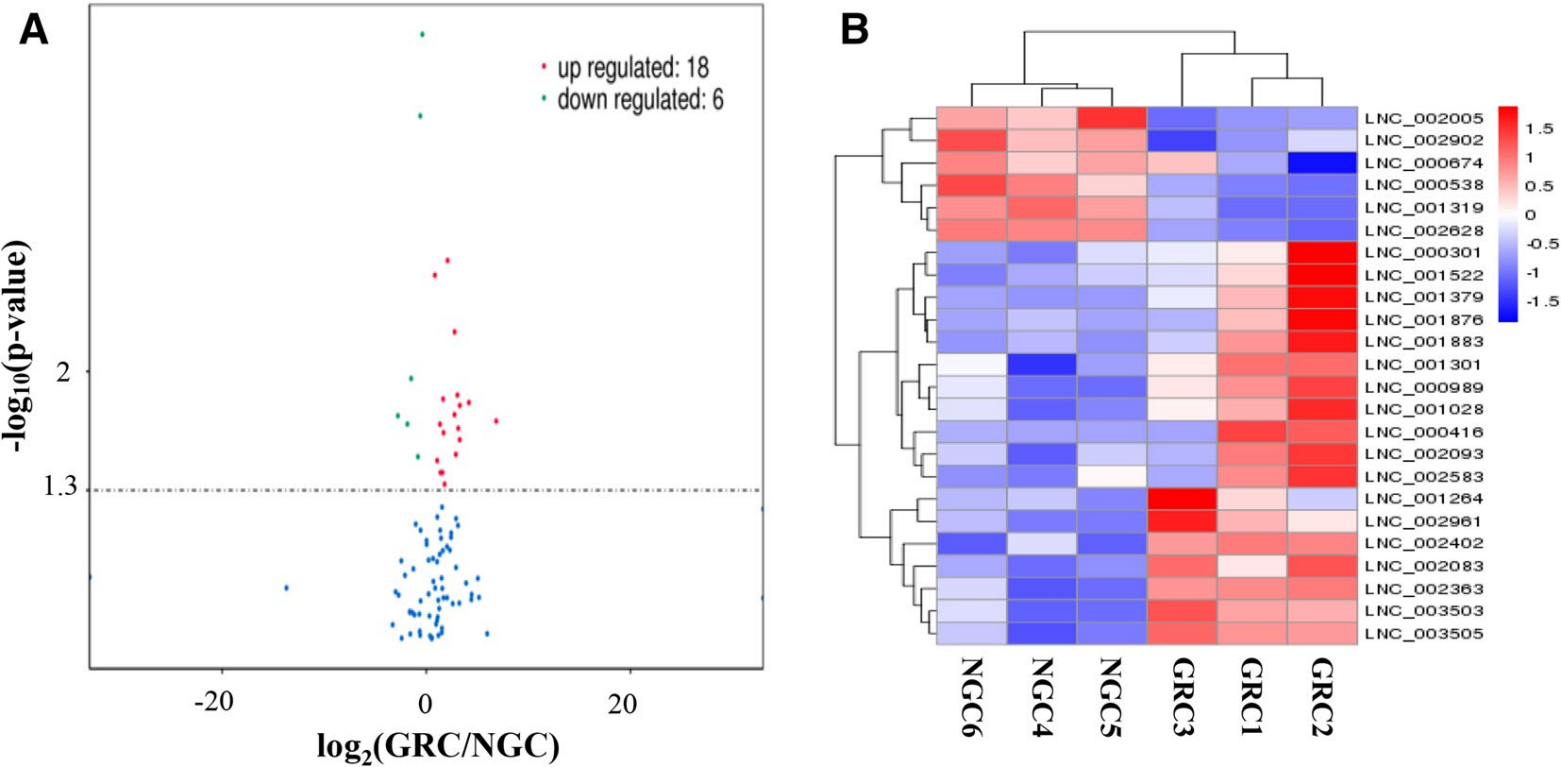
Analyses of differentially expressed lncRNAs in the RNA sequencing (RNA-seq) libraries. (**A**) Volcano plot showing the overall distribution of the differential transcripts or genes, with the threshold set to *P* = 0.05. Red: relatively high expression; Green: relatively low expression. (**B**) Hierarchical clustering analysis of lncRNA expression profiles from 11 libraries with 24 differentially expressed lncRNAs. Data are expressed as fragments per kilo base of exon per million fragments mapped (FPKM). Red: relatively high expression; Blue: relatively low expression.

In our study, 24 lncRNAs were found differentially expressed (Fig. 2 and Additional file 4); the sequence of them was included in Additional file 5. Compared with NGC group, 18 lncRNAs were upregulated in the GRC group and 6 lncRNAs were downregulated (Table 4). For these differentially expressed lncRNAs, LNC_002093 and LNC_001379 were discovered 4 exons; LNC_002628, LNC_002005, LNC_000989, LNC_003503 and LNC_001876 showed 3 exons; all the other 17 lncRNAs had 2 exons, accounting for 70.83% of the differentially expressed lncRNAs (Additional file 4).

**Table 4:** The 24 differentially expressed lncRNAs in the liver of Leiqiong cattle.

Transcript ID	Gene ID	FPKM (GRC/NGC)	log_2_(GRC/NGC)	*P* value
LNC_002628	XLOC_115691	5.82/7.64	− 0.39314	1.04 × 10^–4^
LNC_001319	XLOC_060016	4.58/6.92	− 0.59342	3.14 × 10^–4^
LNC_002583	XLOC_114076	5.04/1.19	2.082334	2.22 × 10^–3^
LNC_002093	XLOC_093077	1.75/9.80	0.8358590	2.71 × 10^–3^
LNC_001264	XLOC_057064	1.57/2.30	2.76908	5.85 × 10^–3^
LNC_000538	XLOC_026579	6.44/1.80	− 1.48464	1.10 × 10^–2^
LNC_003505	XLOC_162058	2.02/2.44	3.048021	1.38 × 10^–2^
LNC_002363	XLOC_104219	6.43/2.06	1.643864	1.45 × 10^–2^
LNC_002961	XLOC_132490	2.06/1.14	4.167938	1.52 × 10^–2^
LNC_001379	XLOC_061866	4.57/4.70	3.283323	1.59 × 10^–2^
LNC_001883	XLOC_086039	2.01/2.98	2.757798	1.79 × 10^–2^
LNC_002902	XLOC_130325	8.01/5.51	− 2.78255	1.82 × 10^–2^
LNC_000416	XLOC_020667	1.61/1.40	6.847665	1.96 × 10^–2^
LNC_002005	XLOC_090526	1.21/4.40	− 1.85589	2.04 × 10^–2^
LNC_002402	XLOC_106316	5.28/2.08	1.340555	2.04 × 10^–2^
LNC_000989	XLOC_045297	1.79/2.04	3.126715	2.16 × 10^–2^
LNC_001028	XLOC_046713	3.01/9.33	1.688786	2.29 × 10^–2^
LNC_003503	XLOC_162058	2.88/2.98	3.272133	2.52 × 10^–2^
LNC_002083	XLOC_092862	1.86/2.51	2.890866	3.08 × 10^–2^
LNC_000674	XLOC_032076	4.33/7.64	− 0.81978	3.17 × 10^–2^
LNC_001301	XLOC_058880	4.53/2.17	1.060875	3.34 × 10^–2^
LNC_001522	XLOC_067835	1.74/8.52	1.580438	3.93 × 10^–2^
LNC_001876	XLOC_085744	1.39/5.31	1.391823	3.94 × 10^–2^
LNC_000301	XLOC_015845	5.97/1.73	1.788629	4.60 × 10^–2^

*FPKM* fragments per kilo base of exon per million fragments mapped, *GRC* growth retardation cattle, *NGC* normal growth cattle.

In total, we detected 114 protein-coding neighbors corresponding to the differentially expressed lncRNAs (Additional file 6). Based on the above targets of the lncRNAs, GO analysis revealed 561 significantly enriched GO terms (*P* < 0.05) (Additional file 7). According to the GO annotation, we screened the GO terms associated with growth and development, including positive regulation of cytolysis, succinate-semialdehyde dehydrogenase (NAD+) activity, cell chemotaxis, regulation of MAPK cascade, and positive regulation of smooth muscle cell proliferation (Table 5). Pathway analysis showed that the co-location genes were significantly enriched in 18 KEGG pathways (*P* < 0.05) (Additional file 8), some of which were associated with growth and development, including Type I diabetes mellitus, chemokine signaling pathway and Toll-like receptor signaling pathway (Table 6).

**Table 5:** Gene ontology (GO) enrichment analysis of the protein-coding genes co-located with the differentially expressed cis-acting lncRNAs.

GO terms	Number of genes	*P* value
**Biological_process**		
GO:0048247: lymphocyte chemotaxis	6	5.87 × 10^–6^
GO:0034341: response to interferon-gamma	6	9.41 × 10–^6^
GO:0070374: positive regulation of ERK1 and ERK2 cascade	7	1.61 × 10^–5^
GO:0043410: positive regulation of MAPK cascade	8	3.36 × 10^–5^
GO:0071347: cellular response to interleukin-1	5	1.22 × 10^–4^
GO:0070098: chemokine-mediated signaling pathway	5	1.70 × 10^–4^
GO:0034612: response to tumor necrosis factor	6	2.94 × 10^–4^
GO:0019637: organophosphate metabolic process	10	5.50 × 10^–4^
GO:0006152: purine nucleoside catabolic process	6	5.72 × 10^–4^
GO:0043065: positive regulation of apoptotic process	5	6.25 × 10^–4^
GO:1901136: carbohydrate derivative catabolic process	6	1.25 × 10^–3^
**Cellular_component**		
GO:0042612: MHC class I protein complex	3	2.43 × 10^–4^
GO:0044420: extracellular matrix part	3	2.81 × 10^–3^
GO:0005615: extracellular space	12	7.18 × 10^–3^
GO:0005578: proteinaceous extracellular matrix	3	3.76 × 10^–2^
**Molecular_function**		
GO:0008009: chemokine activity	7	3.17 × 10^–7^
GO:0042379: chemokine receptor binding	7	4.93 × 10^–7^
GO:0042605: peptide antigen binding	5	1.85 × 10^–6^
GO:0001664: G-protein coupled receptor binding	8	8.09 × 10^–6^
GO:0048020: CCR chemokine receptor binding	5	5.60 × 10^–5^

**Table 6:** Kyoto Encyclopedia of Genes and Genomes (KEGG) pathway enrichment analysis of the protein-coding genes co-located with the differentially expressed cis-acting lncRNAs.

KEGG pathway	Number of genes	*P* value
Herpes simplex infection	9	6.07 × 10^–5^
Antigen processing and presentation	5	5.70 × 10^–4^
Viral myocarditis	5	3.96 × 10^–4^
Type I diabetes mellitus	4	1.23 × 10^–3^
Chemokine signaling pathway	7	1.43 × 10^–3^
Autoimmune thyroid disease	4	2.28 × 10^–3^
Linoleic acid metabolism	3	4.09 × 10^–3^
Toll-like receptor signaling pathway	4	1.45 × 10^–2^

### Combined analysis of the differentially expressed lncRNAs and differentially expressed mRNAs

Among the 114 protein-coding genes co-located with the differentially expressed lncRNAs, nine of them could be found in the DEGs list, including ILKAP, GLTPD2, PSMB6, IFI44, IFI44L, OSGIN1, CASP6, BOLA and B2M. For the 24 differentially expressed lncRNAs, LNC_001319 was located 43,587 bp upstream of PSMB6, which was enriched in the pathway of proteasome; GO analysis showed that PSMB6 was associated with cellular metabolic process, protein metabolic process, catalytic activity and organic substance catabolic process. LNC_001264 was located 79,489 bp upstream of OSGIN1; and OSGIN1 was related to metabolic process, catalytic activity, positive regulation of cellular process and positive regulation of biological process. LNC_002961 was located 24,399 bp upstream of CASP6, which was mainly involved in protein metabolic process and organic substance metabolic process. LNC_000301 was located 12,704 bp upstream of B2M; pathway analysis revealed that B2M was enriched in antigen processing and presentation.

### Validation of differentially expressed lncRNAs and mRNAs

**Figure 3:**
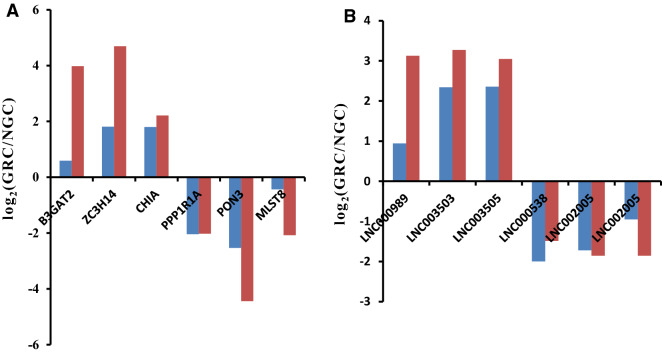
Validation of six differentially expressed mRNAs and long non-coding RNAs (lncRNAs) using quantitative real-time reverse transcription PCR (qRT-PCR). (**A**) The qRT-PCR results of differentially expressed mRNAs were compared with the RNA sequencing (RNA-seq) data. Red: RNA-seq; Blue: qRT-PCR. (**B**) The qRT-PCR results of differentially expressed lncRNAs were compared with the RNA-seq data. Red: RNA-seq; Blue: qRT-PCR.

We randomly selected six differentially expressed lncRNAs and six differentially expressed mRNAs for qRT-PCR analysis. Results demonstrated that the gene expression trend in the two experimental groups was consistent between RNA-Seq and qRT-PCR method, although the fold change of the expression level, to some extent, differed between the two sets of data (Fig. 3).

## Discussion

Liver is an important organ for nutrient absorption and metabolism. However, in the liver of growth retardation fetuses, there are always disorders of growth and development and nutrient metabolism^32–35^. Therefore, in the present study, we performed transcriptome sequencing for the liver of Leiqiong cattle with growth retardation and those with normal growth and analyzed the differentially expressed mRNAs and lncRNAs to clarify their roles in growth and development. In this study, our results provided a valuable catalog of functional lncRNAs and mRNAs associated with growth and development.

Through RNA-Seq, we totally identified 1,124 differentially expressed mRNAs and 24 differentially expressed lncRNAs between GRC and NGC experimental groups, which may have specific biological roles in growth retardation in Leiqiong cattle. Using qRT-PCR, twelve randomly selected differentially expressed lncRNAs and mRNAs transcripts were validated, and the results were found to be consistent with the RNA-seq data. Among the identified DEGs, wnt inhibitor factor 1 (WIF1) and encoding succinate dehydrogenase complex assembly factor 4 (SDHAF4) are significantly differently expressed. WIF1 is an extracellular inhibitor of the wingless signaling pathway that binds directly to Wnt proteins, thereby inhibiting the transmission of Wnt signaling. The Wnt signaling pathway transmits a growth stimulation signal, and the abnormal activation of the pathway can cause abnormal proliferation and differentiation of cells and cause tumor formation^36^. The WIF1 gene also appears as a paternal gene-expressing imprinted gene in human embryonic trophoblasts^37,38^. In our study, the expression of WIF1 gene increased in the GRC group, biological analysis results revealed that WIF1 was engaged in the GO terms including “regulation of fat cell differentiation”, “regulation of cell differentiation”, “cellular developmental process”. Studies demonstrated that SDHAF4 gene could promote mitochondrial succinate dehydrogenase activity and prevent neurodegeneration^39^. In livestock and poultry research, the biological function of SDHAF4 has been rarely reported. In our study, the expression of SDHAF4 decreased in the GRC group, and the bioinformatic analyses revealed that SDHAF4 was engaged in the GO terms including “cellular metabolic process”, “succinate dehydrogenase (ubiquinone) activity”, “organ development”, and “mitochondrial respiratory chain complex assembly”. Thus, we made the hypothesis that SDHAF4 might play roles in regulating the growth and development of Leiqiong cattle; however, the underlying mechanism still requires further investigation. The ACO2 gene can promote the biosynthesis of lysine, generate ATP by participating in the tricarboxylic acid cycle, affect the proportion of muscle fibers, and regulate muscle growth and development^40,41^. For the genes B3GALT1 and UPP2 that we found to be extremely different, there are no reports related to the growth and development of the body, which requires further cytological validation. In our study, the expression of ACO2 and UPP2 were decreased in the GRC group and the expression of B3GALT1 was increased. Through bioinformatics analysis, we found that ACO2, B3GALT1 and UPP2 are mainly involved in Metabolic pathways, Carbon metabolism, Citrate cycle (TCA cycle), Glyoxylate and dicarboxylate metabolism, Biosynthesis of amino acids. The JUNB gene product (JunB proto-oncogene, AP-1 transcription factor subunit) has been described as a growth-inhibiting protein, and JUNB deficient mice showed a retarded growth and a reduction in adipose tissue^42^. The expression of transcription factor JUNB was significantly down-regulated in the model of muscle atrophy induced by diabetes, denervation and starvation. JUNB also plays an important role in regulating the synthesis and degradation of skeletal muscle proteins. It has been reported that knocking down JUNB in mice can significantly reduce the cross-sectional area of muscle fibers, while overexpression can significantly increase the cross-sectional area of muscle fibers and promote muscle hypertrophy in mice. The main reason for muscle hypertrophy is that JUNB promotes protein synthesis and inhibits protein degradation^43^. In our study, the expression of JUNB gene increased in the GRC group, biological analysis results revealed that JUNB was engaged in the KEGG pathway including “osteoclast differentiation”, “TNF signaling pathway”. EGFR is a member of ErbB family. It has tyrosine kinase activity and is an important transmembrane receptor. EGFR was positively correlated with the development of central nervous system and the growth ability of cultured neurons in vitro. EGFR is activated by ligands to initiate intracellular signal transduction. It regulates transcription of transcription factor-activated genes through cascades of binding proteins and enzymes in cytoplasm, and directly participates in cell migration, adhesion, proliferation, differentiation and apoptosis. EGFR is expressed in chondrocyte and can promote chondrocyte proliferation by binding with ligands^44,45^. Overexpression of EGFR in mice can accelerate the proliferation and growth of osteoblasts^46^ and the proliferation of osteoblasts is impaired after knockout^47^. In our study, the expression of EGFR gene decreased in the GRC group, biological analysis results revealed that EGFR was engaged in the KEGG pathway including “regulation of actin cytoskeleton”, “PI3K-Akt signaling pathway”, “ErbB signaling pathway”, “MAPK signaling pathway”. KEGG pathway enrichment analysis showed that PPAR signaling pathway was significantly enriched by the DEGs ACOX2, SCD5, CPT1A, PPARα, ACOX1, RXRB, PCK2, SLC27A5, CPT2, EHHADH and RXRA, playing key roles in the regulation of multiple types of cells metabolism process. As a key member of this pathway, PPARα is involved in lipid metabolism in the liver and muscle, and is a key regulator of hepatocytes growth and metabolism; it has been demonstrated that PPARα could be utilized as a candidate gene for marker-assisted selection for growth in cattle^48–50^. CPT1A gene is reported to be involved in regulating the growth traits in goat^51^. RXRA could influence cells proliferation and survival, influencing animals immune reaction^52^. PCK2 is suggested to play certain roles in modifying the function of liver and the development of muscle^53,54^.Thus, we hypothesized that these above genes were involved in animals’ growth and development process; however, the underlying mechanism still required further investigation. In the next work, we will further verify the function of the genes found in the above on the cells.

Transcriptional regulation could be affected by the activities of non-coding RNA (ncRNA) and transcription factors. Only about 2% of the mammalian genome is transcribed as proteins; 75–90% is transcribed as ncRNAs, the vast majority of which were lncRNAs^55^. In human and mouse, lncRNAs have been reported to potentially regulate muscle growth and differentiation^56–58^. In the present study, we totally identified 3,705 lncRNAs; 24 of them were differentially expressed in pairwise comparison between GRC and NGC groups, which might have specific biological roles in regulating animals growth and development. Studies have demonstrated that lncRNAs could influence the myoblast proliferation and differentiation through regulating the growth related genes; additonally, lncRNAs could significantly affect miRNA biology by acting as a precursor for miRNAs, directly binding to and sequestering miRNAs, or indirectly interfering with miRNA expression and regulation, through which the animal growth and development process might be regulated^59,60^. And, lncRNAs might work as competing endogenous RNA (ceRNA) model, binding to and sequestering miRNAs to prevent their target transcript degradation^61,62^; however, the ceRNA hypothesis remains controversial. In the present study, our identified lncRNAs and their functions were predicted by bioinformatics method, thus further functional experiment should be performed for the validation, facilitating the enrichment of the functional annotation of the identified lncRNAs. For the differentially expressed lncRNAs, most of them were discovered two exons, accounting for 70.83%; just two lncRNAs showed the highest number of the exon, which was four. These lncRNAs were spliced with fewer exons (2–4), which was consistent with previous studies^63^. Their lower number of exons compared to mRNAs could be caused by the weaker expression of lncRNAs, and this made their structure more diffcult to verify^64^. GO analysis showed that the differentially expressed lncRNAs targets were associated with smooth muscle cell proliferation, cell chemotaxis, and regulation of MAPK cascade. The target genes (MAPK8, RPS6KA2) of Lnc_002363 and Lnc_003503 were significantly enriched in the MAPK signaling pathway; this pathway is related to embryonic development, cell proliferation, division, inflammation, cancer and so on^65,66^. Therefore, in addition to mRNAs, the differentially expressed lncRNAs reported here could be considered as important novel regulatory factors involved in the growth and development process in Leiqiong cattle. Among the 24 screened lncRNAs, Lnc_002583 was found located between the differentially expressed genes IFI44 and IFI44L. IFI44 is a kind of interferon-induced gene. The increase of IFI44 expression induces interferon response and inhibits the expression of MSTN gene. Most interferon-inducible genes can regulate cell growth. Some studies have found that IFI44 can inhibit cell proliferation. By transfecting IFI44 into melanoma cells, it was found that IFI44 could reduce the sensitivity of cells to IFN-alpha and inhibit cell growth^67^. The expression of IFI44L was up-regulated in synovial tissue of patients with systemic lupus erythematosus^68^. In order to study the role of IFI44 and IFI44L in the growth and development of Leiqiong cattle, GO enrichment analysis was carried out. The results showed that they were associated with small molecule binding, nucleotide binding, purine ribonucleoside binding, organic cyclic compound binding, GTP binding, viral defense response, stress response and immune system process. Lnc_002583, IFI44 and IFI44L were all upregulated in GRC group; due to the potential cis-regulatory effect, Lnc_002583 might positively influence the expression of IFI44 and IFI44L, exerting co-regulative effects on Leiqiong cattle growth and development. Our identified differentially expressed lncRNAs were all novel lncRNAs, they might act as ceRNAs to regulate animals growth; their regulatory effects in other species are still uncertain, which needs further verification. For the long non-coding RNAs related to the growth and development of the above screening, and the regulation relationship with the target genes, further experiment should be performed to verify the function and explore their regulation mechanism on the growth and development of Leiqiong cattle.

## Conclusions

The present study provided a systematic description of the changes in mRNAs and lncRNAs in Leiqiong cattle under the condition of growth retardation. The data was helpful for further investigation of the lncRNAs function. Generally, our results contributed to basic information to elucidate the mechanism associated the regulation of growth retardation in Leiqiong cattle at molecular level.

## Supplementary information

Supplementary Legends.

Supplementary Information - Additional file 1.

Supplementary Information - Additional file 2.

Supplementary Information - Additional file 3.

Supplementary Information - Additional file 4.

Supplementary Information - Additional file 5.

Supplementary Information - Additional file 6.

Supplementary Information - Additional file 7.

Supplementary Information - Additional file 8.

## Data Availability

Our sequencing data is being loaded to the sequence read archive (SRA) of NCBI. Once the submission ID was obtained, we would include it in our manuscript.

## Abbreviations

lncRNAs: Long non-coding RNAs
RNA-seq: RNA sequencing
DEGs: Differentially expressed genes
ORF: Open reading frame
FPKM: Fragments per kilo base of exon per million fragments mapped
GRC: Growth retardation cattle
NGC: Normal growth cattle
GO: Gene ontology
KEGG: Kyoto Encyclopedia of Genes and Genomes
