# Supplementary material for: Growth retardation-responsive analysis of mRNAs and long noncoding RNAs in the liver tissue of Leiqiong cattle
Source: Sci Rep. 2020 Aug 31;10:14254. doi: 10.1038/s41598-020-71206-4 (PMC7459292; doi:10.1038/s41598-020-71206-4)
Supplement: Supplementary file 1 — Supplementary Legends. [file 41598_2020_71206_MOESM1_ESM.docx]

**Additional files**

**Additional file 1:** The 1124 differentially expressed mRNAs in the liver of growth retardation cattle and normal growth cattle (XLS).

**Additional file 2:** Gene Ontology (GO) enrichment analysis of the differentially expressed mRNAs (XLS).

**Additional file 3:** Kyoto Encyclopedia of Genes and Genomes (KEGG) pathway enrichment analysis of differentially expressed mRNAs (XLS).

**Additional file 4:** Details of the differentially expressed long non-coding RNAs (lncRNAs) (XLS).

**Additional file 5:** Sequence of the differentially expressed long non-coding RNAs (lncRNAs) (DOC).

**Additional file 6:** The protein-coding neighbor genes corresponding to the differentially expressed long non-coding RNAs (lncRNAs) (XLS).

**Additional file 7:** Gene ontology (GO) enrichment analysis of protein-coding genes targeted by cis-acting long non-coding RNAs (lncRNAs) (XLS).

**Additional file 8:** Kyoto Encyclopedia of Genes and Genomes (KEGG) pathway enrichment analysis of protein-coding genes targeted by cis-acting long non-coding RNAs (lncRNAs) (XLS).
