# Supplementary material for: Growth retardation-responsive analysis of mRNAs and long noncoding RNAs in the liver tissue of Leiqiong cattle
Source: Sci Rep. 2020 Aug 31;10:14254. doi: 10.1038/s41598-020-71206-4 (PMC7459292; doi:10.1038/s41598-020-71206-4)
Supplement: Supplementary file 6 — Supplementary Information - Additional file 5. [file 41598_2020_71206_MOESM6_ESM.docx]

>LNC_002628

GCCTGGAGCTCCAACAGGAGCCCAACGAACCCAACGGCGCCCGGGGGTCTCGCGCGGTCGGTTAAGCGGCTCTGCGATCAGTAGAGGCCGGGCTGCAGTCCCTTACGCATCCTCGGGACCAGGAAAGGGAGCGATGAAGGCGCCGGCGCCCAGCCGCTCTGGCCTTCCGCCCGCGGCTCCAGCTCAGGGCCCCCGACCACTTCGGGCCACCTGGAGACCTGGCCGTCGGGAGTATTAAAAGCGCCCCGGCCTCTCGGGAAGTGCGACCAAAGCCGACAGCGACTGGTGTCGAGGAAGAAGGGGGTCGTTTCAGGCTGTGACATGGAAGCAGTTTTCTCGAGGAGGAAAGACGAGTTTTACAGACCGTTTGGTGACCGCGCCTCAGCTCTGCCCAGCGTTCACTCTACAATTGCAGTGAGCACCTGTTATGTGCAGCGATCACAGAGATGAGACCGACCAGAGCCTTACTGTCGAGGAACTGGACAAACAAGAACCCACAGAGGCCCCAGGCTGCTAAAATACATCCCAGAGGTCGACAATCCAGGAGGTGCTTAGCAAAGGACAAGCCAGAGACACGCGGAGCTCATCAGGCTCTGAGCGCCTGGCTTCCCAGGAAGGTGTCCAGGCTGCCACAGTCCCCCTGCCTAGAGGGCTTCGGGGGCACCGAGGACACGTGCCGATTGTACCAGGTGAGCCCTGGGGAGGCAGGAGGAGAGCCGCAGGAGGCACAGTGGTCCGCACCCCGTTTCTTCCCCGCTGCGCACAGACCGGCCTGCTGGTCCTGACTCGGATGGCTTCCCGGACCAGCTCCCTGGCAGTTGGAAGGCTGGGCAGACTCCCACGCGGTCCAGTCCCCACGCGCCCCCTGCCTTCCCTGGGCCCTAGAGCAGGGAGCTCACAGCGCAGAGGATGAGACGGCGGTCTGTTGTCCCAGCGCGTGGCAGGGCCACGCAGCTCTGTTATCTCAGTTTCTGAGGGTCGGGGATCCAGCCAAGACCGGCTGGGGCTTCTGCCTCGAGGTCTTGCGAGGCTGTACCCGGGTGTCGGCGGGGACTGTGGCCCGTGCACAGGCTCCACCTGGAGGGTCCAGGCTCACTCACGTTGGCGGGATTCGGCTCCTCACGAGCTACTAGGGGAACGTCATCCTTTTCTCCATGTTGCCGGAGGGCTCAATCCGTTGCCACGTGGGCCTTTCCAGAGCGCACCTCCCCGCGGGGACAGGCCCCCCGTGACCCCCGTCTGCAATGGCAGCTTGTCCCTTTGTGCTGTTAGATTCTGCTCACCCTGGGGGGCCTCGCAGGGTGCTCATTCAGTGGGGGCCCCTGGGGGCCAAACCCCCGCCCCACTCTGTGCTCTTCCTACCATCCCCCAGGCCTGGGTCTCCAGAGATGGTCATACTGTGTCCAGTGTGACAATTGTGTGTTTTTCTCTCGTTTTAGAAACTGTAGCCTGGGCTGTCGCACCCTGAGATAGATGTACCCAGAGAGTTGTAGGGTGCGCTGTGGACACTGTTCTGTCCATGCCGCCCCTGCGTCAGCCCTCCCCGGGCCTCTGCTTACTAGGTTTTCCCGCCTCCTCCGGGAGACAGAGCCGCTTTCCTTAAGCTCAGGAGCTGTTTGCATTTTTATCAGAGCAGGATCAGGCCCCCAGTCTCCGCGGAGTGGAGGAGCAACAGGGGAAATCCGAGAAGGAAGCATAGGGGCCCGCACAAGCCACACAGTCAAGGCTTGAACCACAGCTAAATCTCGGTGTTGGTGAGAGCCTGGGAAGAGCCTCCGCCAAGGCCTGGGCGCCCCGGGTGCCCCAGGAGGCCGAGCCAGAGGGGCTGGGTGAGGCAGGGCGGCCCCAGCCGCATGGTGTCCCGAAGCTGCATGGTGTCCCTGTCTGGTGTCCCCTGCCCAGGGCCCGCCAGGAGCTGGCACCTCATGCTGTTTTTCTGTTTGTGGGCTGTGTAAGTTAAAAAGGACTGGCTCTTTCTTTACCGGCAAACCTGCCAACCTTGACTGCCCTGGCTTGACAAGATGGAAGGAAAGGGGGAGGGAAGGCGGGAAGAAGGGCCCCAGGCCCCGCCCCTCGCCACCCCTCCCCTGGGCCAGCCCTGGGTCCTGCTTATCCTCCCGGGCTCCATCCAGCCCCTTGGCATTTGACCACTCCACTCTGGGGACACTGTGCCCCAGCACTGACCCCTGGAGACACTCGGGCAAGGGGAGCCAGGCTCAGTCCCGGACCGGAGCAGCCCTCAGGTGGGCCAGCACTGGGAGCAAAAAGGAGAGGACTCAGCCCCTTTTTCCTCGACACGTTATGGAAATTTGCAAGCACAAAGTGGAAGGGATTTTTCACTACACACCTGCACGCCCACTGCCGATGATCCCACTCACGTTTTACTAGACTGTTATATCTAGGCAACCATCTTTTAATTCATTTTATTTTTGATGCATTCAAAATCAATTTTAGCCTCAAATGTGCTTGCCCCTACATACTTAAGCATGTACATTAACTAGAGTTCATTAGTGATAGTCTTTTAATATAAAACAATGAAATGCCCGGCTTGTAAGTGAACATTCACTCTTTTGACAAGTGTATGCATTCTTGTAACTCAGACCCTTATCGAGATACTGAGTGTTCCCATTACCCCAGACTGGTTGACCCCCAGTCCACTCAGAGGCAACCACTCTGTGACCTCCTTGTGATACTGGCACAAAGGTACTAAGTGAAGTCGCTCAGCCCTGCTCGCCTCTTTGTGATCCCGTGGACTGTAGCCCACCAGTCTCCTCGTCCATGGAATTTTCCAGGCGAGAGTACTGGAGTGGGTTGCCATTTTCTTCTCCAGGGGATCTTCCCAACCCAGGGATTGAACTCAGGTCTTCCCCATTGCACGCAGATGGTTTACCATCTGAGCTACCACAGATTTAATACTGGAGCCTAAAGCAAAAGGGAATTATTTTTAATGGCTAATATTTTTTTTCCCAGAACACCGAAGTAGTTAACAACCTGTTGAAAAATGAAAAGTAGATATATCAAAATAGTTTATTTATACAAAAACCAACCAATAATCTTGCTTTCCTGGTTTTAGTGGAAATGAACTCATCAATATTTCCGAAGTCTACTACTTACCTTGTTTTGAAAGGAGAGAATTGCCATGCTAATTTCTTTTGATGAAGTGATCTTAATTTTTCATTTACTGCAGCTTACTGAAGTGTCTTATAGGATGACATGGTGACTGGGAATGCTAACAACCTTCTCAAGGCCATGTTCAAATTGGAAAGTATTGCACTAAACAAATTTGTGAATCAGCCTTAGAAGATGCAAACACTATTTAATAACTGATGCTGCATGCTGCTTAAAACTATATCAGATGGATATATAGGATTTAGGATATTTGAGTCAAGTAAGTGACTGCTTTGTGTAAGAGTTATCCGTAGAAAACTTTTCTCCATTTAAAAATTCAAAATGTAAGACACTTTTTCATATAATCATTTACAATGTGAAAATTAAGGCATCTATAGTTTCTTTAATTTCTATTTGATATGCAGTTTTATAATAATTGACACTCATCTAGTGATTCATTTCCAATTTTTATACTTTAATTTTAAAATTCAAGTGCAGCATTGGTAAGATAGTGGTGAGCATAGCTGCCTTTCATAAAGTTCAAACAGTAGCCCATATCTTTTGCTAAGATTTCACATTAATCAAACATTGAAAGTTTCTGTTATTTCTAAGCAAAGGTAAAGGCCTTCTGCTAAGAGTACAACAATGTATAGTCTTTTTTTTTAATTGTATAAACATTAACTTTTACACTGGTTTATAATAACATCCTACACAGATTGTATAAAATAATGCATTTGTGCCAAGATGCTTTTGATGTATGCTTTTCATTTAATATATATTTCAGTATAAGTGCCTTTGTATATGAAATACCTTGTTTACATTTAATCTTGATCCTCACGGGATATCTGGATGTGTTTTTAATGTAGATTTTAGAACAGACATAAGAATATCCCATCTGTGCATCCATTTTTAAAACTTCACACACAAAATATATATGTATATTATATGTATCCATATACATATATATAAATGCTAAAAAATGGTTAACATTTGATTTCCCAGGGCAGCATTTATGCATATTTTTATGAGCTGAGGAAGCACAAATTTGGCATAATCATATTTGGGGGCAAAATGTCTTATAAACTCTTGTATAACAGGCTATATTTTCTCCATTAGACTATACCTGGCCACAGGCATTTTAAATTTTATTTCCAACCTCTAATTTCATAAGCTAGACTTAGAAAGTTTCATCTTTCATTTCAATGAAGTCAGTACAACTTACTTTATTGTTATGACATTGTTCTTCATGTGAGCAGATCTCGTGGTCAGACATGCTTGTTCACGATGGGCTACATCTTGTGTACAAGCAAAAAATAAAAAGTACTTGCTCCATTATTGACAGTATTTCAGAACTCATGTGATTTGCTTGTAACATGGTATACTTGTTTTGAATTTTATTAGACCAGGCACATGGATTTCCAACAATTTCCTGTGATTCTCTTAAGATTTAAATTGTTTTCTTCAAAGTGTAACATCTAAAACCACTCAATATATTGTGATTTTTGTTTCTTTGATTTTGTCATCTTCTGCAAAACATCATCTATTAAACTGTCAGTTTGAATATTTTTCAGAAACACTTCCCATTTTAGACAGCATCACAATGAGACATCAAATTTTCAAGGTCACAGCTTCTGTCTAAATTTTTCTAATATTTGAAACCTTTTTCATGGTTACAAAAATTTGATTCTGTTGTATTATTGTGGGGAAACATGCACAAAAAATAAAGACACTTGTGAATAAATGAACCAGTCACAGTGAATTTATTTGCCATTATAATAGTAAAAAAAAAATTTG

>LNC_001319

GTTAAATCCTGCGGAGGGAAGGACAGCAGGGAGAAGCTAGCTTCCATGGCCTCCTCCCACCTTCTCCACCCTCCCACCCCCGGCTCTTACCGATGACCAAGTCAGGCTTGGGCCCCTGGAGCAGGTGGTAGGGCCTTGCAGACACCTTGATCTGCAGGTCTCTGCGGCCCCCCTTCTCCTTAGTCCCAGAGCTGAGCCGTGCTGATACACCAGAACCAGGGCGGACAGATACTTCAGGGCCATCCTGGGGAGTCAGAAGTGGGAGAGAGGCCAGTGGAAGTGATGGTGCTGGAAGACGTGTCAGGTTGCTCACCAGGGTGTCAGTTTCACTTCAAAGCTGTGCCTATGACAAACATGGCAGGTTCGAGCCCGAAGGGAGGCAAAGGTGAAGCCTGGCCGGGGACCCCATGTCCGCATCGGGGTCTTGGCTAACTTCACTCCCAGCCTTGGGACCAGATTCTCCAGCTCCCTGAATGAGGGTGGAGACAGCATCAGGTTGGGGGTGGAGCTCAGAAGCCCAGGCCCCCAGGCTTCCCCACCCTCTACCAGCTTTCTG

>LNC_002583

CATTAATCATTAGTGTAGGCACTTAGAAACATAAACCCAGATACTGAAGGACATTGCTAGTCTCATATTACAACAAACCAATAATCAACTATCAATCTCTCTATTGTAAATTTAATTTTATTGTAGTTGAAAATAAACATAAGATTTGGACTTGGTAACTCATAAAAAATGAAAGAGACAAAATTGAATTCAGTTTTTTTTTTCTTTCTTTTTGTTTGTTTGTTTGATATGCAGTTTAAAGAAGGTACACCTTTAGCTTTAGGAGCATACGACGTCTCGGATTCAAAATCTGAAACAGTCAACGGAAACAAACTAAATGTTGGGTCATTTCTGCCCCCTAGTGGTCAATGCTCTAAATGGAGAAGATGCTGCTGCTGCTGCTAAGTC

>LNC_002093

GACTAAAAAAGCTACCTCTTGGCATTGATTTTCAAATTGGATCAAAGTATTAAAAAAAAAAGGGAGAATGGCTCTTAATTTTAGGTTAGGTCGAGAAGGAGAGGTAAGCAAGTGGGCTCAGAATTCTGCAGGCAGGGAAGATGTGTCTAAGGAGCTCAGCTTGGAGGACTGACTCAGTAGGAAGTGGGAGGCTGAGGCCGGGCTGTGAGGGAAGGTCCTGCCTGGGCAGGTGAAGCCTGGTAATAAGATCAGATTACATAATGGGTTTTTTGCAAGGCTGGTTGGCTGTGAGTCGGCTCAGCACTCGGCATTCAAGTCTTCCCTCCCAAATCCACCACCTGGAATCCACCGCCATCCCCTGGGCCTGGCCCACGACGTTGACAGCAGCTCGGAGCGCCCACCTCTCTCATGGCACAGAAAGGGTGACTACTCGGCTTGTTGCCGCGGGTCCCACGGGACGGTGAGGGGCCGGGTCAGGCTTCTGTGCTCCACGTGGCAGGAGTACGGGGCTTGGTCCTCAGGGGGGACCCCCACCACCACCCAGGACTGGCGATGGCTGTGGAGGCCACAGGATGAGACAGAAGAATCCCAAGATGTAAACAGCCTGGATCCCAGAGTCATCCTATGGAAGAGACTGCCTGATGTGGCTTCAGTTTTACACGAGGATTTGCCTGATCTGCATGCAACTTCACATGAGCAAGAAATAATCCATATCTGATGGATTAAGCCACCTTGAACTTTTTTACTGGAGCATAGCCTAGTCTAACTTGACAAATACATGTAGTTAGAAGGTGCTGGGAGGACAATTTGCTAGAAAACTCAGGGAATGACCTAGAGTAGCTTGGCACAGGGTAAGACCAGGATGGATCTCAGAATTCCCAGGGCTGTAACTTGCCCTTTGGTGTGAGGTGGAGACTGGGAGAGGGGGGAGCATTCCCACAATCAATTCCATAATTGATTCATCCTATAACTCACATGGGTGTTAATCATGTTGGAGGTCGTACGGCAAAATCCTAATTCAACTAGTATTTTTGCTAATTAGTTTCTAAATGTGATGATCGTTTAGCTCAGTAGACCTCAAGTAAAGCAGATTACCCTCTGTAACGTGGGTGCGTCTCATCTAATCAGTTGAAGAACCCAAGGGGAAAGACTGAGTCCCCTGAAGTAGAAATTCTGCCTCAGGTTGCCTTCAGATTCAAGACAGCAGTGCCAGCTCTTCTCTGGGTCTCCAGCCTGCCGACCTGTCCTGCAGGTTTCTGACTTGCCAGCTCAGCCCCACAATTGTGTTAGCCAATTCTTAAAATAAGTCTTTTTCTCCAGAGTCCTATTAGTTCTGTTTCTGTGGAGAACCCTGACTGATACAGAGTTTGGTGTAGAGGGTGACATTCTAAAGGTGATTGATTGTTATAAAGTTAACATAAAACATAAAACTTGCACGG

>LNC_001264

CCGCTTTCGTGGTTTGACTTGGCCTCTTGTGCTCCAGTTCTGGCCTTGAGGAAGGCATGTCCCAGAAAGCCGTAGGCCCTGCAGGAATAAAAGACCCACAAAGTGAATGGAAACCCAGCCATGGCCAGAACAGCGCTGAACCTGGCTCCCCTGCTGATGCAGCTGAAATCCAAGAAGGCAAGAACACAATTCAATGGCTTTGAAGATTTTTTGCCTTCTGGTTTTGCCTCAGGAGAAAGAAGAACTTAATATGACTGGAAAAATTAAGATTTCAGTTATACACGATAGAGATAAACTAAAGAATCCCATAGAGTTCAAATTATTGTCTGTGCTCCCAGATGGCATTTTATATCTGTGGTTGAGGGAAGGAGAAATATCAACCTTGCTAATAAGTAAGATAACCAGTTTGAGTGTAATTTCCTGAAATATGTTAATGGATGGTTTGGTTTTCTAAAGTAGTCAGGGGACCTGAATCTGAAATTTGGATGTTCTTTTACAATCCATAGAAAAGACATGACAAGTTGTTCTAATGTGATTAGAAGATTATGTCTGATCACATTTCTCTTTCTAAGCACACAGTCTATGAAATTAAGGCAATTAAGAAGAGATCATTGTTGTTGTAACTTGATCCTCCCACTCCCTAGAATTAATCTGTCCAATAGAATTAAAGGGTTGCTTCCCTCCTGCC

>LNC_000538

CTCAAAAGTAGTGTACAGGGACTTATCCATTCCTGCTTGTGATACTGAAGTAAAGACATAATGTCTGGAGATGTGGCAGCTGTCTTGCAACCATGAAGCAACAAGCACAGAAGGATGGTGAAGTTGAAGGGTGAAAAGGGTCAAGGGCTCTGGTTGAGCCCATTTAATAGCTAAGGCTGTTTCTGCGGCTGCCAGTTACCAGATGTGCTACAAGGAGCAGTAATAGTCGTAAGGACCTTGGCCTGGCACTCAAAGCAATGCCACTGCTGACCTGTAATTCCACGGTTGACCTGCAGACCTCTGCCAGGGGTGGAAAATAAAGACTGAAAATGCTGACAAGGGAGGTGAGGAGAAGCAAGCAGAATCCGGAGAAGCAAGAGAGCACTGAGGAAGCAAAATGAAAGAGAAGAACAAATAAACAGCCTTATAT

>LNC_003505

CACCCACCCCAGCTCCCTCTGCTTGCCCGTGCTTTGACACAGTGGCGGTCATCTCATCACTGACGCGCGGACACGTGGACTCAACACGTGGTCCCCCGTGCAGTCGCTGAGTCAGAGCACATTTCCTGAGCTGGGACCGCGCGCCCATCGCGGTCCTGAGAGGTTCTGAGCCACAGGGCAGGGCCTGCCTCCCGTGAAGCCGAGCTTCCAGCAGGAGAGGAACAAACTGCAACAGGAGCGTAAACCAGTAACAGCACCGCAGCAGCCGCGGGAGGCCAGCTCCACAGACAGGGCAGCTGCGCTTTCATCAGGAGGGGATCCAAGCGGAGGCCTTAAGATGTGCCAGATCCTCTCAGACACAGATCTTATCAGCCTTTCAGCGTCCAGCTGGTGACTCTGGAGTTTTATTTAGTTGATTCCCCAACACCCGCTCTAATTCAGGATCTAAATCAACAGCCCTCCTGTGTGACACGTGTCCAGTTCGGGTGTGTGCTTGTCACTTTACAATGATGAGGAAAACCATCACATAAACATTTGGCAGAGAAACAATTTTCAATTTGCTGTTTTCATCGCACCTGAGCTTAGGCAAGAGAGTCTCGGTTTCCAAACCGTAGGCGTGGTCTCAGCTCAGAAGCAGCACTGCGGGCCGTGAGTTCCGTGATTTCAGTGTTTCCCACAAATCTTATCACGCAGGGTTTCTTTCCTGGGGGAATGGGAAGTCAGCTCAGCCCCTGACCCTGACCTCAGGGCTCCTGGGCTCTGCTCTGGGCTGAGGGACGGACTGTGAAGTTGGAAACTTTGATCACATGGACACGTCAAGGCCTGCAGACCTCTGCTTCTTTATCAGGTAAACTGATATGGACTTGGAGACCGGATCGATCGGGTTTTGTTTTTAGTCGTAAATAGCAGTTGAAAAGGTAGATGCTCAGACTGAAGAAAAGATTTTTTTTAAGAGAAATTAAGACCAGAGTTAGGCTCTGCACATAAATTTAAAACACATTTTCAGAGCCTAAGATTTTTGCTAACAACACTCTGGCCTAGTCACACCAGATCCCCGGTGAGACCTAGAAAACATCCAGAGAAACCCGGAATCACGTGGGACTTGATCCAAAAGACTGACGGGAACCACAGTGGAAGCATAATGTCTAAGCCCGTGAGAAGGTTAATGATGAAATAATCAACGTTGGCCTCACGGCCGCCAGATGAGCAACTGTCCCTGACGTGTTGGACCAGAGGAGGCCAGAGACCAGCTCCTCACCAGGCCCCAGGGATGCCAGCAAAACTCTGCCCTCCAACAGCTTTCTTCCTGATGAGAGAGGAAACAGAAGAAACAAGCAAATCACACAAAGGATGTGACCATCACAGGGCACCGAGTGCTGGGGGGACACAGGGAGGCCAAGGGGGCCGTGGAGGGCAAGTTCAGACAGAGGGAGAGGGTCTCACGGAGAAGATGCTCTGAAAGAGCTGGAGGGCCAGGGAAAGGGGGCGCTGCGGGGCGGGGGGAGGAACTGCAGAGCAGGGAGGGGACTGCGTAGCAGGAAGGACTGCATGAGCTCATTTCCTCTTCCCAGAGCTTCACTACAGCTACCCCCACCCCACCCCAGCCAGCCCCCCAGCCCCGCGCAGAGTAGGGGCTTCCCGCGAGTTTGCGATTCTCAGAACGTGCCACCAGAGGGCAGTGTAGGTCCTGCAAGCGTTGACAGGGTCACTTCCGCCCCCTGCGCCCCCTCCTAGATCACACGCTTTCCTTGTGGGGGGCCGTGGTGGGGGGAGGGCAGCTGTCCCAGATGCTACCTCTGTTCCTTATGCCCCGGCACGAGCTGCCGACCCGAGATCTCCGCAGAGCTGGGCGTCCGTCAGCATTGCCACGAGCTCTGGGAAGCTCATGACGCAGAAGCTCATGGACACACCATAAACCACACAGGGCCAAAGGTCAGGATCCTTTCAGTTCTTGACTTCACGTTGGCGTAGCTCTCACACACGGAGAGAATCTCTCCTTAAATGGCACCCCACTCCAGCATTCTTGCCTGGAGAACCCCACGGACGGAAGAGCCTGGTGGGCTGCTGCCAGGGTCCAGCCCCGGCTGATCCAGGGTATTCGAAGGAGAGACGGCATAGGCGAAGATCAGGAAACAATTGCTTAATTAAACGTTAATTAAGGATATAAAGAGTAATAGAATGAGGATAGCTCAGTGAGGAAATTCAGTGGAGAAAAGAGGCTGAATAATTCAGCCAGAAGGTGAGAGAAAGAACGACATGGGGAGACCAAGTTTCGGTGAACAAGGCCCGCACTTTATTTTCCAAAGTAGTTTTTATACCTTAAGTTATGCATAGAGGATAACGGGGGAAGGGGTAGAGTCCTGCAGCAAGCCAGGCTTTCTTCCTGCAAACTTATCATATGCAAAAGCTTAGGTGATTTGCATCATCTTCTGGCCCGGAGGCCTGTTAACATTTTAAGACCCTTTCTTCAGAAAACTTATTTTTCTCTAAAGATGATTGGTCAGGAGCCACCCTCCAAAAGCATTAGATAAAGTTGCATTCCTACAGAGCAAAGGTGTGGTGGGCTATAACAAGAAAAAGAATTAACTCAAGGGTCCCAGGTTACAAACATTAAAGCTACTACTTACACCAATTATATTAATCAATACACTGCCAGGGACACAGCAGGTAAGGGATATGGAAACTTAGCAGCAAACACTGGCCCAACAAGTGAAAATCCCTTCACCAATACAATTTCTAATCAATCTTTTAACTACTCAAAGGAATCTGTGTTTAGACAGTTTAGAACATCTCCTGCCTCTCACAGTTGGGAGGCTCTGAACAATCACATGTGGCTGGAAAAACCTATTCAGGCAGGCTAGAGGATTTCCAAAGGAGTTTGTAGGTTAAACACTGTCACACCCAGGAATTATTAACTGGAGCTGTAAGCTAACTCTTTTTTCAGAGAGAGGTGGTGGGGGACAGCCCCCCGTAAAGTCAGAGGTGTAGGTGAGAGCACAAAGCAGAAAGTAGGCAGACTCTGGTTTTGGGGGTAGATGCTCGAGAATTTCCAGGGGGACTCCTGAAGCTCGATCCCGCCTTTGCGTATGCCGAGCTTCCTTACTCATGACCTTTGTCATGGGCGGGGTTCCTCACGCTGGCTCCCGGCAGTGATAGAATTCCATTTGAGCTATTCCAGATCCTGAAAGATGATGCTGTGGAAAGTGCTGCACTCAATATGCAATATGCACTCAATATGCAATATGCCGGCTCCCGGCAGGCTGCAGTCCACGGGGTCGCAGAGTCGGACACGATTGACCGTCTGAACACGACACTTCGCTGATCCGCATCGCTGTATGGCAGAAACCAACATAACACTATAAAGCAACTTTCCTCCCATTAAAAAATACATTAAAAAAATAAAACAAGATATTAAAAAACTCTTGTAGGGAATTCCCTGGTGGGCCGGTGATTAGGACTCAGGTTTGATCCCTCCTTCGGAAC

>LNC_002363

TCATTTCTCGATGACGGGGGAATCTCGGGGTTGTTCTCGAGCGGCGGCCCCAGTGTGCGGTTTCTCACGAGGTACGACGGCGAGGTCAGTGAGCCTCTCGTGGGGCGCCAGGGAAGTCGGGTCTCCATGCGAGTGGCGAGGGGGAGCGCGTCATTGCTCCCGAGCCATGGTAGGGGAGGGCATCTGGCCTCGAGACGTGTTGAAGAAGGTCTCTCGAGGTCTTTCCCGGGTTGAGGCAGGAAACCCTGGGTTCCCTCGACTTGTGCAGGTGACCTCAGGGGGCTTCTCATGGTGGCTCTGAGAAGCCAGGGAAAGTGGA

>LNC_002961

GAGAGGGATAGGAGTCTAGGGTAATTCCCAGGTTTTCTCTTGGATAATGAGGTGATCGGTGTTGACAATGATTGCAGAAATAAAGGGAAGAGAGGAATAGAAGTAGGTTTAGTCAGGAATATCTGGAAGCTGTGCTGTCTATGGGAGTCTGGCAAAGATAAATAGTAGGAAAATATAAAGTAGGAAATAAAAAATTCAAAATCAATCAGAGGTTTGAAAGTAGTCAGAAACCCTACTGAGAGAAATCACACACCTGAATTTATCCTGATGGTCTAAAACCTGCAATTTCACTTAAGTGGATTTCTTTCCATCTTCTCCAGTGCTTCCTCTTTGTGTGCCAAGTAATCTTACATTCTAGCCACAAAGGTCACTTGAAAATCTCTCTCCCTGATTACTATGGTCTGAAAATTTGTGCTCCACTGTGAAAATTAATAAAGGGAAACCTCACTAAAATGCAGTTGGGTGATCAGAAAGGGAAGCTCTCATGCATATGCCCTCTATATATCAATCAGTGGTACAGATCCCAACAGGAAGAAACTACTTTGCATTTTCAGGAGGAAGTGACACTACTTTACTACCACCAGCAAAAGGAAAAAAGATTTTCTTCTTGTCTGGTAACAGCTCAGCCAATGAGAGAGTGTCACAAATTGGCCAGTGAAAAGCCAGTATACATCAAGCTCAGAGTTTTCTCCAATGGCCTTTTCATTTATAACAGGCCCCTCCCAACTTCCCGATATCCTCTATAAAAGACTTTCCTTTCCTTTGTTTGCAATGTGGACTTGGATGTGGTTCAGGACAATGGCATGTCTTGAATTGTAATTCTTTGCTGCTCCAGAATAAACCTGTTTTATTGGGAAAATAACTGGCTGTTTTATTGTTTTACATTGACCCTCCATACTTATATGTTGAAATTCTAATACCCAGTGTGATGGTATCAGTTCAGTACAGTCACTCAGTGCTTTCTGACTCTTTGCGACTGCATGAACCACAGCACACCAGGCTTCCCTATCCATCACCAACTGCCAGAGTCTACCCAAATTCA

>LNC_001379

GTTGACTGAGACTGATTTGTAATTTTATTGTTAAGCACAGATCCAAAGTCTTGCTGTCTAGTACAAAAGATAGTCCCCAAAGTTACAGATTTATTGCCCTCTAGGATTTGAATTTGTTTTTCTCTAACAAACATTTCAGAATGCCATCTCCAATAACTATATCAACTTCTGTTCCTCAGATGATCTTTGAATCGGTTTAGTGATTAATGGGTTAAATACACATACTAAGTCTGTATTTGAATGCCATGTTTTTAGCTTAAAAAAGTTTACACTGTGATATCTTGTTCAGACCCTCCCCCCCAAAAAAAGCCCCACGAATCCAAAGTTCAGCAATTCCTACACAATCTTTTCCCTCTAAAAGTTTGAGTGAAGTCAAATTGGATACTCTTGATGAATGTGTTGTGCTTGTAATTAGAACAGAACAGTAACTGTTGCTTTTTAAAAAGTAATTCAGGCCACGGCCCTCCAATGAGGACGGTGAGGCAAGAGAGACGGAGAAGAGGGGGCCCGGTCCAGGCGCTTCCGCTCAACCAATAGCGATCTCAGGTCCTCGACGGCTGACCCCAGCCTCAGCTCCGACTTGCATTTCTGAACTCCCTCATTGTTAGGATCAGCACAGACACTCTGCCCCTTCTTGGTGATGAAGATGACAGCTGGCCGGGAGCACGCACTGCTCGTCTCTATATAATTTTCCATGAATACACATCGGATGTTTCGTGGGGTGTAAGACATGCAGCAGTCCGTGGGCCGGTAAAAACCTCCTTGAGACAATGAGTGGTAATTCTGTTCCGGACTTGAGAGTTCAAACTTCAGGTTTGACATCACGTCTTTTACAAGTGCAGCCTGGGCAGCGAGGATGAGGAAGGGCAGGGCAGCTATGGAGGTCTTCATCTTCCTGGTGTACAGACGGTGGACCAGCCTGAGGACTCTGGGGCTCTCTCGGACTTCCC

>LNC_001883

GTCCTTTCCAAGGTAAATGTAAAGTATCACTTCCATAGAGGAAGAAAACTGGTTCTGAAAAATGTAAATACAGTCAAAACAGCTCAAGACAGCAGAGGAGCGGTATGTGGCTCTCACGTTCTCCCACAAATACATCAAGATACATCCACCTGGGACAATTCTCGCGGAACATCTACTGAACACCGCAGATCTCAGACTTCCAAAATGGACAAATCTGACCTTGGATTAACCATCTACCTTGGTTCCTCAGAAGATCTGATAAAGGAGTGTCAGCTGTGTTACTAGGCCATATCGTTCATGGTAACAATGCCCCATCACTGCTCAACAGGCTGCACTGGAGAGACTGGGAATGGATCGTGATGCCTGGAGGGGAGCCACAGTTCTGCCTTTGCCGAAGACAGTGGCTCTTGGAACTGACTGCTCCTTGCAGGAGCCTGGGATGTGCCCCTGGGATAGTGACAAGAGATACTGGCACTAGAGGGCCTCAGGGGGTCTAAGCCAAGGTGGATCTCGCACCCACCTTTGAGAATATCCTCTCCTCGTACTGAGCGTCACACAGGGGGCCACAGCACCCCCTGGATGGCTGGAAGGACTTCTTTTGAAGAGAAAACACCAAATACTGCCCTTGTGAATTTCCTATCCTTTTGCTTTCTAAATAGACTGGGACCAAACGTGGCTTATCTTGAGAATCTAAGTATTAATATCTGTGGGATGCTAAGAGGTCTCTCTGGTGGGTGCTGCAGAATCAATAAGTATTTACAGAAAAGACCTCTTCTGATAGTGCAGAGAGGACACAATGCAGAAACTGTATTTACTTCCTATAAAGCAGAGGGAACATTTTCCTGTAACACTTGTCCACAAGTCAAGTCTGAAGATGTCAAAATAATGTTAAATGAATCACTGTGTGCTGGGCTTCATTAATTACTATTTAAATATGTATTTTCAGAAGACACACTCTAAGACACAAGTCCTAAATATGAATGCTATGTTAATAATACCACAATAAAACTGCATAGTATATTTCACTCGCTTCTTGTGAGCAAAGATAACATCAATAGGAATATATAAGTACCATAAAACATCAGAGTTAAAGGGAAACCTACATCAAGGAAAGTTCTGTTTTGTGTAATATATACTAGCTTTCCTCCTTAAGCTTATTTCAAATACATACATCTGCTGCAGAAAATTCGCAAATAAAACTATTTGGTCACAAATTTTAGAGAAAACCATGCTAATATTTTACTAAATGCCCTTCCAGACTTATTCTTACTAAATACATTCACGCTACATACACTTCCTTGCAGTCTCTCCTCTCCTTTCAACTGAACAATGTATGATGAAAATTTTTGCATCTTTGTATTTATCTGTATCATTTTTAATGGGATTTTTTAATTAAAAAATGAGATCATACAAACTGGTTTTTTAAAAAATGTTTACCTTGTAAAGGAACTCACATTATTTCAAATAGCATCAACTGATTTTAAGTGTTCTTGGTAGACAATGAGTTCACCTGCAAAGAACCTTCTTTCAATTTTAACATCTGACTTTGTATTGTTTAATTAAACTGGCTAACTCCCAGAAGAGTGAATAACAGTGACAATTTCTCCATGCAGTTCTGGGAGTTGTCTTGTTCTGTATAGTTCATAAAGAAAAGCTATGTTAAAAAGCCTAAATAATAATGACAATATATAATGGCTAATATATACGCTGAACGCTTACCATGTGTGAGGTATTATTTTTAAGGGTTTTACATATTTAAGTTCACTTAAATCTCATAATTCTACAAGGTAGGTACTATTACTCCTAGTTTCAGTTCAGTCGCTCAGTCGTGTCCGACTCCCTGCGGCCCCATGAACCCCAGCACGCCCGCCTCCCTGTCCATCACCAACTCCCGGAGTCCACCCAAAC

>LNC_002902

CTCTAAACCCCTCTGGAAGTGAGTTGCTGGAGACCAGAATCTAATTATTGAAGCAAGATTCCAGCCTCCAAGACCTTCCCTGAATTCCAAAGGGTGGATTCCAACTGTTACTAATCAAGGGAGGAACAGCCAAGAAACCACCCAAGGCAAGATTGAAGGGACCAGAGAAACTAATCAAGATTAGGAAAACCACCACCTCAGACCATGCACACCTGAACCTTGTCAGCAACGGCGCACTGAAGTATTGAATTCTTGGGTTGGGAGGATCCCCCGGAGAAGGAATGGGCTATCCACTCTAGCATACTTGGGCTTCCCTGGTGGCTCAGATGGTAAAGAATCTGCCTGCAATGCGGGAGACCTGGGCTCGATTCCTGGGTTGGGAAGATCCCCAGGAGGAGGGCATGGCAACTCTAGTATCCTTGCCTGGAGAATCCCCATGGACAGAGGAGTCTGGCGGGCTACAGTCTGTGAGGTCACAAAGAGTCGGACACGACTGAGTGACTAAGCACACACACAGTTTCAGTTGCAGCACGTGGTTATCTTCCACCTTCATTGCAGTATGTAGGGTCTAGTCCGCTGACCAGGGATAGAACTCGGGCCCCAAGCATTGGGAGTGCAGAGTCATAGCCACTGGACCATGTCAAACTCTGTCTTTGAGATTCAATTCAGCACAGGAGCACAGAGACCAAGCTTTTGGTATCATGGGCATAATATAGATAAGAGGACTTCTCTGCTGAGAGTCCCAGGAGGCCAAGATCAATGTGCCCATAGGGATGCCTTCCTTTCTGAAGGCATCAGAAGGAAAACTCTGCTTCCAAGCTTATTCAGGCTGCTGGGAGAATCTGTTCCATGAGAATGTAGGTCTGAGGTCCCTGTTTCCCTTCTGGCTTCAAGTTCAGGACCATTCTTAGCTCCTGAAGGCTGCCCTTAGCCCTCAGCTGTTCATTTGTCTGCCTTTTTCTTTTGCCTTCCTTTTCTGCTTATGAGGGCTCAGGTGATTACATTAGGGCTGCCCCAGACAATCGAGGATTATCTCCTTATTTTAAGATCAGTCTGTAAGCTGATAGGTAAGGGGTCTCTGGAGAAAGAGAGCCAGGCAGGCTTTCTTGACTTAAGAGAAGCCATTTTGTGATCTAAGCCTTGCTGCAGTGCTTGCCTTTGAACAGGTCTCAGTCATCAATGATCTTAAGGGAACAAAGGAATTCAGGGACAGAGAAAAGGCAAATGAACAACCCTATTGCACTGATGGGGCTTCCCAGGTAGTGCTAGTGGTAAAGAACCTGCTGCAGGAGACGTAAGAGTGCAGACCATCCCCGATTGGGAAGATTCCCTGGAGGAGGGCATGACAACCCACTCCAGTATTCTTGCCTGGAGGATCCTGTAGACAGAGGACCCTGGTAGGTACAGTCCATGGGGTCACAAAGAGTCGGAGAAGACTGAAGCGACTTAGCAAGCACACACGCATTGCATTGATAATTCCTCCTCAAGGCTTATACATAAGAATATATCTGAGTTCTGCAGGAACTAAGGCAGAGCATGCGGGATGGAATGATGATGCTGACCCTCCTGACTTCAATCAACTAAAGCTGGAACTCTGTCAACTATGCCCCAATCGTACACTGAATTCTCCTCTGCTCGAGCGCCTTCATGAATATGCATGTACCTTAGCTTAAAACTTACTCAGTTTTGCTGCTGGAGAGACACTGATTTGGGAAATATCCCTGGTGTCCTACTTATTTGCTGCAAGTAATAATAAACCCTTTCTTCTCTCAGTCTTTGGCTTGGTTGTGTCTATTGGCTCCACATCCAACAAAAGGCAAGCCTAGTTTTCAGGTGACAAGTTGCACGTACGACAAAACACAATCACAGAAGTGAAATCACATCCACAGGAGGCTGGGCGTGAAATCCTGGGGGAGTGACTCTCTCAGCCACTATATGTTTTAATTTCTCTCAGGATGGACACCCAAGATTGGAGTTGCTGAATCATACAGTTGGTATGTTTAATAAAACTCCCAAACTGTTTTCCAC

>LNC_000416

CAATCTCCTCCCTTTGCAAACCAGAGCAGGGAGACCCAAGCAGTCTACTTCCGAGGCAGGCCCTTCCCCGTGGCTGAGGCAGGGAGGGTCTCCTCATGAGGACGTGGCTCTGCGACTTTACTTTCACTGGAAACGGAAGCTGTCTGTGTTTCCACCTTGCATGCTTGTCGGGGGATGTAGGGGCAGCGTCTGGGGTGCTGACCCCAGCCATCGACGTGAGACGCTGGCCCTGCAGTGCAGCCCAGCCCCTCAGTGGTGAATCAGTGCATGTGGACACAGGTACCAATATCCCCTTTGCTTTCTTTTTATGAAGAACTCTGTCTTTTAGGGATTGTACTTACATGCTACTGTCAGGGAGATCTTCCACCCATCTCAGGAACTGAAGGATAAATACTCAGCAACGTTTCAGCACAGTTGCCTCTTTTGTTTGTTATAAAACAAAATACAGCGGGCCAGTTTCCCAG

>LNC_002005

GAACAACTCACATGTTCCCTGGCGTCAAACGTGATGGCTCCTAATAACTGCATGGGGAAGGAACTTGTGTATTTAATCCACCTCCAGAACAACTCATGTTCTCTGGCGTCAGACGTGATGGCTCCTGATAACTGTGCGTGGGGAAGGAACATGTTGCTCAGGCCCAGGTTGGCGTGAGGTCTGCAGTCAAGAGACCCACAGCCCGAGTCCTCATCTTCCTGAAAACACAGAGGACACGTGAATCCTGGACTGCCGATCTCACCGCTCCAGCTTCAGACCAGCAGCCCCACGGTGCTTCCAGGGAGAGGGTCAGTCTCCCACCTGGTACCTTCTTCCCAGGTTCTGCATCACGCACCCCGGAACGTCTGTAATTAGTCATGAAACCCACATGAGCTGCCTGCAGAAAACTCCATAATGGGCAGGCTAAGCTGAGAAACGGATGGTGAAGCAGCGGGGTTTGGGACCGTCACCCGTCAGAGCTCCGACAGATCACAGACCTCACAGTCACCGAGGGCGGCTCCAGGCAGAAAAAGCCAGGAACACACGTGGCATTGAGTCCAGGGTACATTTGTCAAAGTCTTCTTCAACACACGTTGTGTGTGCGTGTGTATGTATGCTAGTGAAGTGAAAGGCGCTCAGTCGTGTCTGACTCTTTGCAACCCCATGAACTATACAGTCCATGGAATTCTCCAGGCCAGAATACTGGAGTGGGCAGCCTTTCCCTTCTCCAAGGGCTCTTCCAACCCAGGGATCAAACCCAGGTCTCCAGCATTGCAGGCGGATTCTTTACCAGCTGAGCCACCAGGGAAGCCCGTATGTATGCTAACCCGTGTCCAGCTCTTTGCAACCCCATAGGCTGTAGCCCTCCATGCTCCCCTGTCTATGGGATTCTCCAGGCAAGAATACTGGAGTGGGCTACCACTCCCTTCTCCAGGGGATCTTCCTGACCCAAGGATCAAACCTGCATCTCCTTCGTCTCCTGCATTGGCAGCGGATGCTCTACCACTGAGTCACCTGGGAAGCCCAGATGTTGTGAAAGCAATGAAGTTAAGTCTCAGAGATTCCAGGCTCTGAAGTGATTCTGCCACATAAGCCCACAAAAATGGCACCTGGGGCCTTTCCTGTCTTCATGAACGAATGAGGTTTTACTAAAAACGAGTATAAATATTTGTACGGGTTTTCATCTACCTTCACATTAAAATCTGCTTGAAGTTCCCATCACACACTTTATTTTGGTGTTGCTAATCCTATGTAAAAATTTCCATATAAATACATGTATTTTACTGCTAGGACTGTTACTTAATGTGGCTTTAACATGACACTTCACTGCCCTTTCAGAAAGGCCGTCTTCCAAGTCAGTACTGCTATGTCCTTTTTCTTTTCACATAACACTTTCCAAACACCGTCTGAAATCAACAAGGCTAGCTTCTAATACCTGGTTCAAATCCTTCTCTCTTCAAAAATGAGTCTTGACATACAGAGATAATGACAGAATAAAGTAATCATCATCTGCCTCTTAAAACAGACAGCATCTTCCCTACAGCCGTATAAATGATCCTTCAAAACCAGTGATGCTTTGGGCTCGAGTCTGAAATATAATTTATCCTCTGCTTCCAGGAAGACGGTGCCAGATGGGACCCACCTTGTAAACCCCTGGTCTTAGCTCGTCAGCCCAGGGTTATTTTGTTTTCAGATATCCATAAGGATCCGAATAAAAACAACGGAAGCACACCCTGACCCCCACCACACTCAGAGAAACCTAGCCGCTAAAACACGAGAATCATTTGTGTCTAAGTTCCTGCTTGATGTGGTCTTTTTAAACCATGCCCCGCATCCTGGCTGGTAAAGGATAGACTTGGACAGTGGTTGTTGCTGCTGTTGTTCAGTCGTTAAATTGTGTCCAACTCCCTGCGACCCTGTAGACTGCAGCACGCCAGGCTTCCTGCCCTTCACTGTCTCCAGGAGTTTGCTCAAGCTCATGTCTATCGAGTTGGTGATGCCATCGAACCATCTCATCCTCTCTCGCCCCCTTCTCCTCCTGCCCTCAATCTTTCCCAGCATCAGGGCCTTTTCCAGTGAGTCAGCTCTTTGCATCAGGTGGCCAAAGGACTGCAGGTGACTTGTGCAGAATTCAGACTCTCCTGGGTGTCAGCTGGATGGTGATGATCAGGGTTAGTGGAACCAGCCGATCGGAGATGCAGCAGACACTACCCTGACCTCCATGCGGACGACGGGAGATGAGAAAAACTTCTTCTAA

>LNC_002402

CTCCTGGCAATCTTGATTCCAGCTTGTGCTTCTTCCAGCCCAGCATTTCTCATGATGTACTCTCATATAAGTTAAATAAGCAGGGTGACAATATACAGCCTTGATGTACTCCTTTTCCTATTTGGAACCAGTCTGTTGTTCCATGTCCAGTTCTAACTGTTGCTTCCTGACCTGCATACAGATTTCTCAAGAGGCAGGTCAGGTGGTCTGGTATTCCCATCTCTTTCAGAATTTTCCACAGTTTATTGTGATCCACACAGTCAAAGGCTTTGGCATAGTCAATAAAGCAGAAATAGATGTTTTTCTGAAACTCTCCTGCTTTTTCCATGATTCAGCGGATGTTGGCAATTTGATCTCTGGTTCCTCTGCCTTTTCTAAAACCAGCTTGAACATCAGGAAGTTCACGGTTCACGTATTGCTGAGGCCTGGCTTGGAGAATTTTGAGCATTACTTTACTAGTGTGTGAGATGAGTGCAGCTGTGGGGTAGTTTGAGCATTCTTTGGCCTTGCCTTTCTTTGGGATTGGAATGAAAACTGACCTTTTCCAGTCCTGTGGCCACTGCTGAGTTTTCCAAATTTGCTGGCATATTGAGTGCAGCACTTTCACAGCATCATCTTTCAGGAGAAAGATGATGTCTTTTGGATAGTAACCTCTTATTGGTCATATTATTTGCAGCTATTTTCTCCCACACAGTGGATTGTCTTTTTGTTTTGTCAGTGGTTTCCTTTCCTGTGCAAAAGCTTTTAAATTTAATTATGTCCTATTTTATATTTGCCTTTGTTTCTTTTGCCTTAGTAGACATATACCCGCGCCCCCCTCCCCCCCAAAATGGCTGCAGTTTATGTCAGAATGTTCTGCCTATGTTTTCTCCTAAGAGTTTCAAGGTTTCTTGTCTCACATGTAGGTGTTTAATCCACTTTGAGCTTACTTTTGTGTGTGATGTGAGAAAAGATCCTAATTTCATTTGTTTACTGAAGAGACTGTCCTTTCCCCATTGCGTATTCTTGCTGCTTTTGTCGTAGATGAATTGTCCTTGGAAGTGTGGGTTTACTTTTGGGCTCTCTGTCGTGTTCCGTTGAGCTGTGTGTCTGTTTTGTGCCAGTACCATACTGTTTTGATTATGGTAGCTTTGCAGTGCACAGTTGAAGTTAGGGAGATGATTCCCTCCAGCTTTGTTCTTTCTCAGAATTGCTTTGGCTATTCCGGACCTTTGTGGTTCTGTATATTTTAGGATTATTTGTTCTAGTTCTGGGGAAAATGTCATGGATATTTTGATAGGGATTACATTAAATCTGTAGATTGCTTTGAGTAGTGTGGTCATTTTAATAATATTAATTCTCCAATCTGAACATGGGATTGCTTTCCAATTCTGTATCATTCCTTTGTCAGTGTTTTATAGTTTACAGAGTATAGGTCTTTGACCTTCATGGTTAAGTTTATTCCTAGATATTTTGTTCTTTTCAATGCAATTTTTAAAAAAAATTAAAAAATTTTTAACTTTTATTTTATATTGGAGTATAGTTGACTTACAGTACTGTTTCAGTGTCAGGTGTTTAGTAAAGTACTTCAGTTATACATATACTTGTATCTATTCTTTCTCAGATTCTTTTCCTATGTAGGTTATTATGGAATAGTGACTTCTCTGTCCTGTGTAGTATGTCTTTGTTGATTATCTATGTTATACATAGTAGTGTATATATGTGGATTTATCTCTCCCCCCAGTCTTTCAACTTTGGTAACCGTTAGTTTCTTTTCAAAGTCTGTGGGTCTGTTTCTGTTTTGTAAATAAGTTTATTTGCATCTTTTTTTTTTTTAGATTCAACATAGAAATGATATCATATATGTCTTTCTCTGCCTGACTTTCTTCACTTAATATGATAATCTCTAGGTCCATTCGTGTTGCTGCAAATGGCATTATTGCGTGTCCTTATGTGGGAGCATCCGTATGCGGTCTGCATGTGCCAGGCTTTGGTTGGAGAGCTTGATCTGCAGTGAGCACAGGTCATGTCTTCACCCTGGGCGTGCTGGCAGCTGTCGCCTTGGTGGGAGACGGGGCTGGAGATTGATGGGTCAGGTCCAGGGCCAAGTGTGAGCCAGACCTTTTCCTCTACTCAGTGGTCGTTATTGCCCTGTTTGGGATGTGGTTGGGTCCTAAGGTGCTGGAGCAGAAACCCTGATGGTTGGGTCTGAGCTGGTTCCATTTCCTCTCAGTGTGAATTCTCCCCCCTCCTGGAAGGGGCAGCTTCATCCCAGAGGGGAAGCAGCACTAGAGCAAGAGATGTTGGAGCAGGCCATGGGTGTGGCCTGACAAAGTGGGGCCCTCTTGAGAAACTGACCAGAGCCCTGGGAAGTCTCCATTTGCTTCCTCTGCCTTGTTCCCAGGAGTAGGCACGTATGTGCACGTGTTCTTCATGAGTGGAGTCTCAATTTTTTTGAGCTGTCTTGTAAGTAATACTGGTTTTCAAACCACGTAAGGGCACTTGTCTTCCTGGTGTTGGACCCCAGGGCCAGGGTATCAAATATGTGGTTTGAATCCTTCATGCCCCAGGGAGGGTCTCCCAGCCCAGGATATCTCTCTCATCTTCTGTGTCCCATCCTCGGATTACAGGTACTGACTTGATCACTTCTCCTCCCTTCTTTGCTGACTCTGTGTGGATTTTTACAGTCTTTGTGTAGGAGAGTCTTTCTGCCAGTCTTGTTTGTTTTCAGTGAGAGTTCTACATGTTGCTGTGTTTTTGATGTGTTCGTGGTGGAAAGTGAGCTCCAATGTCCTTCTTTGCCATCTTGATCTCTTCTTCTTTTGCGGGGGGGTGGGGGGAATGGTCACTGGAGGAGACAGTGCTGCTTGGGTGCTGGTGACACTGTGGTTTTTCATAAGCATCCTTGTTCACTGTTGGGCTGCACCCTGCAGGCAGGCAGTCCCTGTAGCTGCCCAGCTTCAGGACCAAGGGGAGAGCCCAGAGGTATGGATGGGGGGATCTTGTATGTCTGAAGCAAGGTCCTGGAGCGACAGCCCACCGCATGCTACGGATGGTGGGCAGGACATGCCAGCAG

>LNC_000989

GAAATGCCTGGGAAGAGTGCCAGACCCTTCCTTCCTTCCTTCCTTTCCTTCTTTCTTTTTTTCTTTCTTCCCCCTCCTTCCTTCCCTACCTCTCTCTCTTTCTCTCTTCCTCACACCCTCTCTTCCTTTTTCCCTCCCTTCCTTTCTTTTCTTCCTCTCCTTCCCTTGCTCTTCCTCTTCATTCGCCCCTCCCTCCCTCTCTTTCTCTCACCCATTTTAAGACTACCTACTGACACCATTGGTGTTACTGCTCACAGAACCTGATCCCCCCACTTCCACTGCAGTATTCTATCCTGACTTCTGGAAGGGGCCAGTGGAGACCAGACAACACGCTGTGCAATCCATCCTTTTCTTGTTGGAGAATTCAGAGTAAAGAGATATGATCTCATAATTGCATGGAATTACACCTGAGATATCAGATGTCCTGTGCTTACCTTCTACGTGCTCTGCTGGAGGGAAGCTAAGATCCTTCATTCTTTTCAGACAACTGGTTCTTCAAAGACGCTGACAAACCAGATAGTCCAGAACTGCACACTCTGGAGCTAATTCCACGAGCAGCTGAGACCAGGATGGATGGATTCTTGGATACAGATAGGGAAGTAGAGAGAGGAATGGAGAGGCTGCTGAACCAGGAGCACTTGTGCAAAGTAAACATGAATTCTGAGGTGATGATAGACAACACAGAACTTTAAAGGTGCAACCAAGGAAATGGAAGCTAAAAGCTTGCAAGTGGCCCTTCCTGACCTCCCTCACGTGTCTCCCCTTTCCTTTCCTTTTTTTTTTTCTCCCTTTTTCCTTCTCTTAGCATCTTGTCCCTTTCTCAATAATTAAGTCTGTAATTCATGAAGGTCAATAGAGCAAAGGAGGTGTGTGTGAGGAGAGGTGGCCCAGAGAGGGGACTCAGAGCTCAGGATTCTATGCATATTCTAGGTCCAGATGGGGTGTCAGAGCCGATAGAGGGAGGAAGTCCACATGGGGGAGGAGGGGTGGAGACTGATTCTATACAGGGCATGGATCAAATAAGTAAGTATACTTAGAAAAATGGGATCTAGGTTTCTCACTGTAAGAGAAAGGCATTGCAAATATGGAAATAGAGAAGATGAGAAAGAGCCCTGTGATATTGGATTGAAATTGAAGGTGTTGGTGTGAACTCAAAGGTGTCAATAAAAATACACATGGAAATGAATGTGTGTGTGTTGACACATTCGTGTATTTTCTAGCTCTGTCTGCCGAGAGGTTCTTGGTGCAGCAGTAGCTCCATAGCCATGAGCACACCTAGTGCCCAGATCGTGGTTTCTAAGTACCATTCCCCACTAAAAGCTCCTTGGAGAAGCAGCTGGTTCTAGGGCTAGGACAGGGTACGTAGAAGTCGAACCTAGAACATCTATCTGTGTCAGAAAAAAATATGGAAATGCTCAAAGGATGACAGGGAAATGTCACAAAGACACAGAAGTCAGCTTGAAGGGGCTCAAATATAACACAATTTGAGCATAAAAAGAATTGTAACGGATTACAACCCATTGAGGGCTTCCCAGGTGTCACTGGGGGGGTAAAGAATCTACCTGCCAATGCAGGAGATGCAAGAGACTCAGGTTTGATCCCTGGGCAACCCACTCCAGTATTCTTGCCTGGAAAACCCCATGGACAGAGGAGCCTGATGGGCTACAGTCCACGGGGCCACAGAGAGTCAGACACAACTGAGAGACTGAGTACATACATAACCCATTGAATAAAACAGAAAATCATGAATCCATACTGAATTAAGGACTTGATAAGAAATGGG

>LNC_001028

GGGAGTACCACTCAGCAGCAAAACACACAGTCTTTACAAGAATACATGGAATATTGAACAAGTGCCACATTCTAAGCCATAAAACATGTATCAATAAAATTGATAAATCCAAGGCAGACTGGTCAGGAAGCAAAAAGAGAGAAGATATGAATTATCAGCATCAGGAATAAGCATCACTATAGATCCTACAGCTAATCAAAGAATAAGAAAGTCACAACTGTAGAAGTTAGTAAGGTAAAAAATACTGTATTTTTTAAATTTATTTATTTTAATTAGAGGCTAATTACGTTACAATATTGTATTGATTTTGCCATACATCAACATGAATCCGCCACGGGTGTACACATGTTCCCAATCCTGAACGCCCTCCCACCTCCCTCCCC

>LNC_003503

CTTTGACACAGTGGCGGTCATCTCATCACTGACGCGCGGACACGTGGACTCAACACGTGGTCCCCCGTGCAGTCGCTGAGTCAGAGCACATTTCCTGAGCTGGGACCGCGCGCCCATCGCGGTCCTGAGAGGTTCTGAGCCACAGGGCAGGGCCTGCCTCCCGTGAAGCCGAGCTTCCAGCAGGAGAGGAACAAACTGCAACAGGAGCGTAAACCAGTAACAGCACCGCAGCAGCCGCGGGAGGCCAGCTCCACAGACAGGGCAGCTGCGCTTTCATCAGGAGGGGATCCAAGCGGAGGCCTTAAGATGTGCCAGATCCTCTCAGACACAGATCTTATCAGCCTTTCAGCGTCCAGCTGGTGACTCTGGAGTTTTATTTAGTTGATTCCCCAACACCCGCTCTAATTCAGGATCTAAATCAACAGCCCTCCTGTGTGACACGTGTCCAGTTCGGGTGTGTGCTTGTCACTTTACAATGATGAGGAAAACCATCACATAAACATTTGGCAGAGAAACAATTTTCAATTTGCTGTTTTCATCGCACCTGAGCTTAGGCAAGAGAGTCTCGGTTTCCAAACCGTAGGCGTGGTCTCAGCTCAGAAGCAGCACTGCGGGCCGTGAGTTCCGTGATTTCAGTGTTTCCCACAAATCTTATCACGCAGGGTTTCTTTCCTGGGGGAATGGGAAGTCAGCTCAGCCCCTGACCCTGACCTCAGGGCTCCTGGGCTCTGCTCTGGGCTGAGGGACGGACTGTGAAGTTGGAAACTTTGATCACATGGACACGTCAAGGCCTGCAGACCTCTGCTTCTTTATCAGGTAAACTGATATGGACTTGGAGACCGGATCGATCGGGTTTTGTTTTTAGTCGTAAATAGCAGTTGAAAAGGTAGATGCTCAGACTGAAGAAAAGATTTTTTTTAAGAGAAATTAAGACCAGAGTTAGGCTCTGCACATAAATTTAAAACACATTTTCAGAGCCTAAGATTTTTGCTAACAACACTCTGGCCTAGTCACACCAGATCCCCGGTGAGACCTAGAAAACATCCAGAGAAACCCGGAATCACGTGGGACTTGATCCAAAAGACTGACGGGAACCACAGTGGAAGCATAATGTCTAAGCCCGTGAGAAGGTTAATGATGAAATAATCAACGTTGGCCTCACGGCCGCCAGATGAGCAACTGTCCCTGACGTGTTGGACCAGAGGAGGCCAGAGACCAGCTCCTCACCAGGCCCCAGGGATGCCAGCAAAACTCTGCCCTCCAACAGCTTTCTTCCTGATGAGAGAGGAAACAGAAGAAACAAGCAAATCACACAAAGGATGTGACCATCACAGGATCCTGAAAGATGATGCTGTGGAAAGTGCTGCACTCAATATGCAATATGCACTCAATATGCAATATGCCGGCTCCCGGCAGGCTGCAGTCCACGGGGTCGCAGAGTCGGACACGATTGACCGTCTGAACACGACACTTCGCTGATCCGCATCGCTGTATGGCAGAAACCAACATAACACTATAAAGCAACTTTCCTCCCATTAAAAAATACATTAAAAAAATAAAACAAGATATTAAAAAACTCTTGTAGGGAATTCCCTGGTGGGCCGGTGATTAGGACTCAGGTTTGATCCCTCCTTCGGAAC

>LNC_002083

TCTGGCCTCGAGACGTGTTGAAGAAGGTCTCTCGAGGTCTTTCCCGGGTTGAGGCAGGAAACCCTGGGTTCCCTCGACTTGTGCAGGTGACCTCAGGGGGCTTCTCATGGTGGCTCTGAGAAGCCAGGGAAACTGGAGGTGGGAGGGGCCTCTCGGGACTCCACTGCGTTTGGTGCATTGGAAGAGGGCCTCATCTCCAGGTGAGGCAGGAACCGCAGGGTACCTCTGATTTCAGACTCCGATCGCAGGGTCCCTGCAGACTGGGGACAGGAGAGTCAGGCCTCGTCTTGGGTTGAGGCATGGAGCTCCGCTTGCCTCTCG

>LNC_000674

CTTTGATAAAGCCTCTACAGCCCATAGACTAGTGTCCGATAGACAGAACTTCTTTCTTCTATAAAGGAGCTGAAAACACCTGGGGGGCCTCAGAAAATTAGGACTTCTGGGGCCTTGTTTCTTTCTTTTCTGGCCCCATGCCTTCCAGGAGCACAGGGCATGGTAAGCACCGAACACCTGCTCAGGGAACCAGTGCTCACCGGCTGTCTTCCTTCCCAACAGGATCCTAGTTCCCTGACCAGGGATCAAATCCCAGCATTGGCAGCATGGAGTTTCAACCACTGGACCTTCAGGGAACTCCCATGAAGACATTTTAGAGATGAGGAAGTGAGGCAAAGGAGACACTTAGCAAGTTGTTTCCTGAGGCCAAGAAATGAATTCAGTCACCTGACTTCTGGACTGGATGTAACTATCAGGCTGAGAAACTCAGGAATGAGAGAGTTGGATCATTTGGAAACCTGGGCTTTAATCATTGGGAAGTAGGAATACTCACACCACATCCTCAGGGTTCTTAAAGGATATGGCAAGTAAATAATGAACATAATGCATTCGAGAATGGCACCTGTGATAAGTAGTGGGTTGTCTCTTTTATTTGAGATGACTTGATGTTTCTGGAGCATCCTTTAATGTTACACCCCTCCATGACGCTATTGCATCTTGTAGGATGCAGGAGACTGTAGGTCAACTGTCTTGAGTCAACCCTCTGCCCCAAATAAGTATGAGTATGTTCATGGATCTTTTTATTGCAGCCACATCTACCAGTTCTTTGGAAACACCTGGAAAATTTACTCATTATCATGTCTCTATAAAACCCATTAAAAACTAGTGAAACTCAGCAATCTCAAATGAAACACTTTGTGAGCTGTGCTTGATTTGAACGACTTTAACTATTAGTAACTTTCCCACCTGTCTTCCTTTTTCCACTAGGCAAAATAGATTATATACTGTAATGTGATAACTTATTGTTCTAATTTGAAAAGTTGCTTTTCACTTGTTGAAATTGTTAGAAATGTATGGGTTGGTATTCTTTTCCTATTTTCCGAGTAGTATCAACTATTTTCATCTAGCCTACACGTATTGGAACACACTCCAAAGTATAATATCTTTCTTCTAGAGGGAGGGGAAAGTGTG

>LNC_001301

TTCTGAATGAATATGCATCATGGGAGAGTAGCAATTAATTGTATGGGAAAGTGGGAGGTTATTCTGTAGTGGAAAGTGTTCTGCCACCACCCTTTTTAGAGTTGAGCATTCTTTTAAATAGTCCTCATGGTCAGAATGTTCTTATGGCAAATGGAAGAATGCGCTATGTCAGATTTCTTATTACTAAGTAATTTATTTTGAAAATAAGTGTCTTCTCCCCATACTCTTTAACTCTGTGTTCCAGTGGAAGACCTTGACACAATCAAATATCAGTGTGGTGCATCTATAATATTTTTTACTTGCTTTCTTATTAACAACAACTAGGAATTTTTTAAACCTCAAAGCAAATTTTCAAGATGTAAACACTCTTCTCTGTACACCAATCCTTGGCCAGCTCTGATCTGGCCTACTGATAGGTTGGCCAGCATATTAATGTCATTCTGCTGAAGATTTTAAGAATTGGCCTGGATCTCTAGAGGACTGGACTGCCTTAGCTTGATCTGCCTCCTAGCTTGCAAAAAGTGACTTGTATTCAAAAGAAATTAAAATGTCAAAATCTAAAAAAAAAAAAAAAAAA

>LNC_001522

AAAGGATTAGATTCATTTATACAATCTTAGTTCCCCGTTAAAATTTTTGTATACTAGATGTTTCCTGCATGGTTTGTCTCCTCAGTCATTGTCCACAGGACTCTGAGAAGCTGTAGGAATATGCAGGCGCAGACTCAAGGCCCAGCTTCATCTCCAACGTTGTGGAAAGGGACTGTACGTCACTGAGCAGAGCCCTACGCTCCCGTTCGCCACCTGGACAGAGCCCGGGGAGAGTGACCTCCCGG

>LNC_001876

AGGGCCGCCCACTTCTGGAAGGTTCCGTCCCCTGAAGGCCTGGTTTCCACAAGCTCCATGTCCTGGATCTGGTCCTCCCCATTGCGTTGCCAGGTCAGTGAGATCTCCTCAGGGTAGAAGCCCAGGGCCCAGCACCTCAGGGTGACCTCACGGTCAGAGATGGGGTGATGGGTCACATGTGCCTTTGGAGGGTCTGCGCGCAGCAGCGTGTCCTTCCCGTTCTCCAGGTATCTGCGGAGCCACTCCACGCACCGGCCCTCCAGGTAGTTCCTGCGTCTCTCCGCCTCACCTGCCGCCTCCCACTTGCGCTTGGTGATCTGAGCCGCCGTGTCCGCCGCGGTCCAGGAGCGCAGGTCCTGGTTCAGGGCGATGTAATCTCTGCCGTCGTAGGCGTCCTGCCTGTACCCGCGGAGGAAACGCCCGTCCGGCTCCACGTCGCAGCCGTA

>LNC_000301

TGAATAGAGAATTTTCGGTAGGAAGTTGTTTCATCACATTCATGATGACCTTCTATGCTATGTTCTGGATCCCACTGACATTGAGAAGTCAGCTGTTCTTCCTTTGAGAAGCTGCACACAATGTTTTACCATTGTTTTAGTGAAACAAAATATATAGTAAAACCAGGCACAACCAAGTTTCTAACAGATATAGGAGATAACATAAATTTTATATGGCAAGATGCTTCATCTACAATCTCCATAATTCTAAAAATTAACAAATTTCCATAACTCTAAAAATTATCTTTCCAAAATTTTCCCAAAATTGTAACCAAAGTTACAAAATTGGAGGCTTACTTTAACGTGCCTAAGTATCTTATTACCCAGTTTTGTATGCAGAAGACACCCAGATGTTGATGTTAAATCTGAAATTGTCTTCCCCACCTCTAAGATGCCAGCACCTCCACTGCAGACACCTGCCTAGGAAGGTCAAACATGGACATGTAGCACCCAAGGTAAAATTATAAAGAAGGTATTTGTGGTAACATGAATATGACTTCTGATTATGTGCATAAAGCATAAAAGCATATCAGTATTAAAGAGCAGGGAAACAGAATTTTCCAGATATTATACCAAATGAAGAATGTTCAAATCTCGATGGTGCTGCTTACAGGTCTCGATCTATGGAAAAGAAAACGGAGAAAGGAAACAGTTAAGTTTGACTTCAGA
